# Yersiniosis in New Zealand

**DOI:** 10.3390/pathogens10020191

**Published:** 2021-02-10

**Authors:** Lucia Rivas, Hugo Strydom, Shevaun Paine, Jing Wang, Jackie Wright

**Affiliations:** 1 Christchurch Science Centre, Institute of Environmental Science and Research Limited, Ilam, Christchurch 8041, New Zealand; Lucia.Rivas@esr.cri.nz; 2 National Centre for Biosecurity and Infectious Disease, Institute of Environmental Science and Research Limited, Upper Hutt, Wellington 5018, New Zealand; Hugo.Strydom@esr.cri.nz; 3 Kenepuru Science Centre, Institute of Environmental Science and Research Limited, Porirua, Wellington 5022, New Zealand; Shevaun.Paine@esr.cri.nz (S.P.); Jing.Wang@esr.cri.nz (J.W.)

**Keywords:** *Yersinia*, yersiniosis, *enterocolitica*, *pseudotuberculosis*, foodborne, New Zealand

## Abstract

The rate of yersiniosis in New Zealand (NZ) is high compared with other developed countries, and rates have been increasing over recent years. Typically, >99% of human cases in NZ are attributed to *Yersinia enterocolitica* (YE), although in 2014, a large outbreak of 220 cases was caused by *Yersinia pseudotuberculosis*. Up until 2012, the most common NZ strain was YE biotype 4. The emergent strain since this time is YE biotype 2/3 serotype O:9. The pathogenic potential of some YE biotypes remains unclear. Most human cases of yersiniosis are considered sporadic without an identifiable source. Key restrictions in previous investigations included insufficient sensitivity for the isolation of *Yersinia* spp. from foods, although foodborne transmission is the most likely route of infection. In NZ, YE has been isolated from a variety of sick and healthy domestic and farm animals but the pathways from zoonotic reservoir to human remain unproven. Whole-genome sequencing provides unprecedented discriminatory power for typing *Yersinia* and is now being applied to NZ epidemiological investigations. A “One-Health” approach is necessary to elucidate the routes of transmission of *Yersinia* and consequently inform targeted interventions for the prevention and management of yersiniosis in NZ

## 1. Introduction

Gastrointestinal infection caused by the bacteria *Yersinia enterocolitica* (YE) and, less frequently, *Y. pseudotuberculosis* (YP) (collectively referred to as *Yersinia* in this review) is a common illness in ruminant animals and humans. Yersiniosis has been a mandatory notifiable disease in New Zealand (NZ) since 1996 and all notified human case data for yersiniosis are collated in EpiSurv, the NZ national notifiable disease surveillance database, which the Institute of Environmental Science and Research (ESR) operates on behalf of the New Zealand Ministry of Health.

The incubation period for yersiniosis is typically 4–6 days, and generally under 10 days [1,2]. Yersiniosis in humans is described as typically causing diarrhea, vomiting, fever and occasionally abdominal pain in children under 5 years old, while older children and adults are more likely to experience abdominal pain as the predominant symptom. Sepsis may occur in immunocompromised individuals [2]. YP is more likely to cause mesenteric adenitis and septicemia than YE [2].

Yersiniosis can also give rise to an array of other, more uncommon clinical manifestations and sequelae. Necrotizing enterocolitis has been described in infants. Reactive arthritis affecting the wrists, knees, and ankles can occur, usually 1 month after the initial diarrhea episode, resolving after 1–6 months. Erythema nodosum can also occur, manifesting as painful, raised red or purple lesions along the trunk and legs, usually resolving spontaneously within 1 month.

Based on epidemiological information, the majority of human yersiniosis cases are considered sporadic with no identifiable source. In addition, there is a paucity of local information on source attribution, meaning that there is currently no evidential base for interventions to reduce disease incidence.

Unlike other enteric bacteria, YE is psychrotrophic, actively growing at the food storage temperatures routinely used to minimize bacterial proliferation [3]. Foodborne transmission is the most likely route of infection but baseline data on *Yersinia* from foods and the environment is lacking as food surveillance is not routinely performed and thus the etiology of yersiniosis in NZ remains unclear. This review outlines the current landscape of yersiniosis in NZ and the actions required to identify reservoirs and sources of human yersiniosis in NZ.

## 2. Human Clinical Yersiniosis Is Increasing in New Zealand

The NZ case definition for a confirmed case of yersiniosis (including YE BT1A) is a clinically compatible illness accompanied by laboratory definitive evidence of either (a) isolation of YE or YP from blood or feces, or (b) detection of *Yersinia* spp. nucleic acid from feces [2]. Cases may have *Yersinia* isolated from multiple specimens but are only counted once in the notification data.

**Figure 1:**
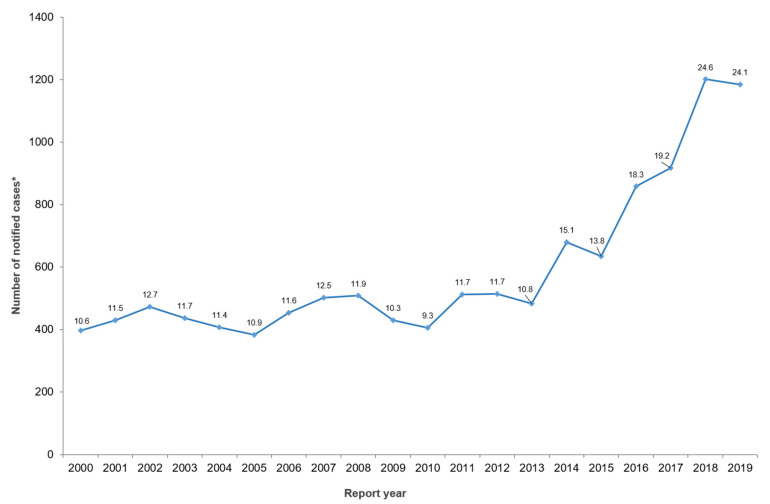
Number of Yersiniosis cases notified in New Zealand by year, 2000–2019, as recorded in EpiSurv. * Value above each data point is the rate per 100,000 population for each year.

Annual notifications of yersiniosis in NZ were relatively stable from 2000 to 2013, with approximately 500 cases per year (rate 9.3–12.7 cases per 100,000 population) [4] (Figure 1). In 2014, 682 cases of yersiniosis (15.1 cases per 100,000 population) were notified, with the increase in cases for that year attributed to a large outbreak of YP involving 220 cases [5]. From 2015, the rate of human yersiniosis in NZ has significantly increased, with a peak of 1202 cases (24.6 cases per 100,000 population) in 2018 and stabilizing in 2019 at 1186 cases (24.1 cases per 100,000 population). The current rate of yersiniosis observed in NZ is high compared to other industrialized countries. However, caution must be taken when comparing data as notification systems, case definitions and testing regimes may differ between countries. The European Union/European Economic Area (EU/EEA) notification rate in 2018 was 1.6 cases per 100,000 population with Finland, the Czech Republic, Denmark and Lithuania reported as the countries with the highest rates of 9.6, 5.9, 4.9 and 4.9 cases per 100,000 population [6]. In the United States of America (US), the notification rate in 2019 for *Yersinia* was 1.4 per 100,000 population [7]. Yersiniosis is not notifiable in many Australian jurisdictions due to the decline in reported incidence and lack of identified outbreaks prior to 2001. The last published rate for yersiniosis in Australia was in 2004 and was reported as 1.3 per 100,000 population [8].

**Figure 2:**
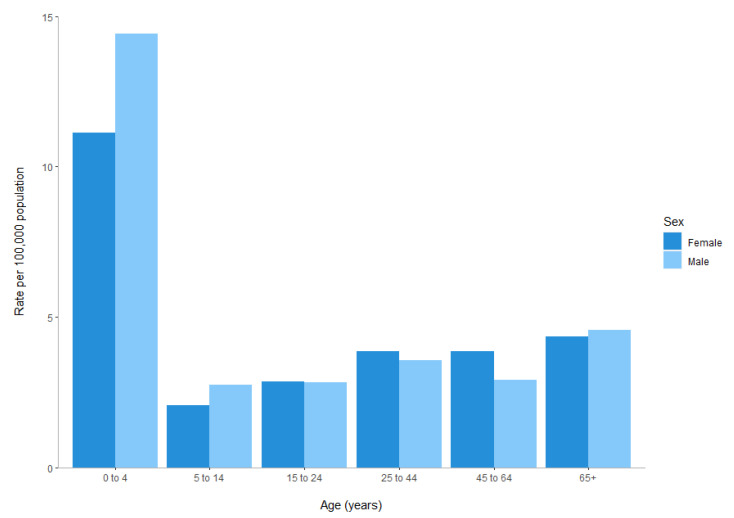
Age and sex distribution of yersiniosis cases in New Zealand, 2012–2019 (rate per 100,000 population), as recorded in EpiSurv.

In NZ, between 2012 and 2019, children aged 0–4 years had the highest notification rate of yersiniosis (children 0–4 years old only represented approximately 6% of the population in 2018 [9]), with neither sex disproportionately represented (Figure 2). The age and sex distribution observed for NZ is also consistent within the EU/EEA in 2018 [6]. Information of co-morbidities or pre-existing conditions of those notified with yersiniosis is not collected as a part of surveillance activities in NZ.

In NZ, Annual Surveillance Summaries stratify notified cases of disease according to the following ethnic groups as used by Statistics NZ [10]: European (including New Zealander), (indigenous) Māori, Asian, Pacific peoples, Middle Eastern/Latin American/African (MLAA) and Other. From 2009, when Asian ethnicity was first reported in NZ Annual Surveillance Summaries, this group has shown the highest notification rates for yersiniosis. In 2018, ethnicity was recorded for 1095 (91.1%) of NZ cases and the ethnic group with the highest notification rate was Asian (40.7 cases per 100,000 population). High notification rates for people of Asian ethnicity are not observed for other notifiable enteric infections in NZ (data not shown). In contrast, Māori have the lowest notified rates of yersiniosis (14.1 cases per 100,000 population) followed by Pacific Peoples (16.4 cases per 100,000 population). However, these two ethnic groups also show lower case rates across other enteric notifiable diseases [11]. The reasons for these differences between ethnic groups is as yet undetermined. In the US, it was observed that there was a higher average annual rate of yersiniosis amongst African American and Asian children compared to children of European descent [12]. In NZ, the Asian ethnicity group includes people originating from a large geographical area incorporating many countries and cultures. More detailed work is required to further investigate these findings before conclusions can be drawn.

**Figure 3:**
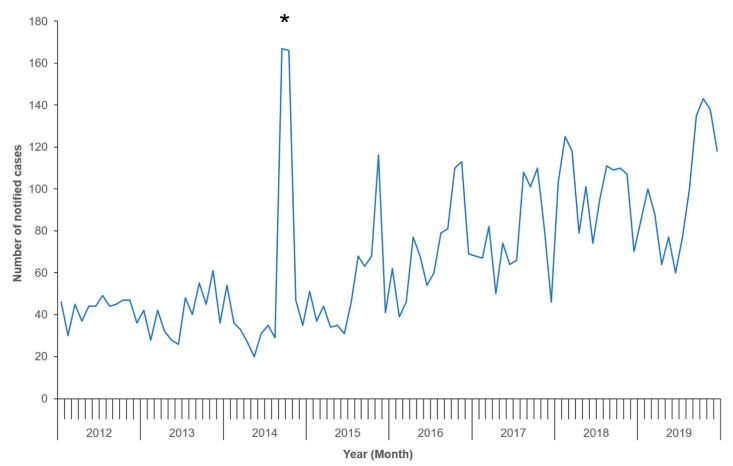
Number of notified yersiniosis cases in New Zealand by month, 2012–2019, as recorded in EpiSurv. * Indicates a peak of notified cases attributed to a large-scale outbreak of *Yersinia pseudotuberculosis* in 2014.

Since 2014, notified yersiniosis cases in NZ have been noted to peak between August and November, which coincides with the spring season (Figure 3). Summer peaks (January–February) were also observed in 2018 and 2019. In contrast, yersiniosis in the EU/EEA did not have a clear seasonal pattern in 2018 (as seen in previous years), despite a higher number of cases reported in May and June [6], coinciding with the spring–summer seasons.

In NZ, between 2012 and 2019, the proportion of hospitalized cases was consistently approximately 12% of annual case notifications. The exception was in 2014 when 22% of cases were hospitalized many of these cases were due to a large-scale YP outbreak [5]. The majority of cases over this eight-year period were diagnosed from a fecal test but in 89 cases (<1.0%) *Yersinia* was isolated from other body sites such as blood, aspirate, or biopsy indicating invasive disease. These extra-intestinal isolates comprised 19 YP (6.6% of all YP received for typing) and 70 YE (1.4% of all YE received for typing). This is consistent with previous findings that YP is disproportionately associated with more severe disease [13]. In the EU/EEA, in 2018, 29% of notified yersiniosis cases (1873 cases with known information) were hospitalized. Three of 3862 cases with known outcome were reported to have died, giving a case fatality of 0.08% [6]. As notification requirements differ between member states of the EU/EEA, it is likely that more severe cases would be disproportionately represented in data from this region.

The burden of disease depends on the severity and duration of the primary gastroenteritis, any non-gastrointestinal symptoms and any post-infectious sequelae. The burden of disease caused by yersiniosis in NZ was previously estimated as 93 (37–161, 2.5 and 97.5 percentiles) disability-adjusted life years (DALYs) [14]. However, due to the lack of NZ-specific data, these estimates incorporate the use of overseas data for under-reporting multipliers and rates of sequelae. Reactive arthritis, including Reiter’s syndrome, may occur a few days after initial gastrointestinal symptoms and may persist for weeks or even months [15]. A case series of 60 reactive arthritis cases from Dunedin, New Zealand identified an antecedent YE infection in eight (13%) cases [16]. A study from the USA reported that symptoms consistent with reactive arthritis were self-reported in 12% of yersiniosis cases, compared to 5% of controls [17]. Similarly, 12% of cases from a YP O:3 outbreak in Finland met the case criteria for reactive arthritis [18], while 12% of 351 German yersiniosis cases reported symptoms consistent with reactive arthritis [17]. However, a Dutch study only identified reactive arthritis in 6% of 261 yersiniosis cases [19]. It has been suggested that the presence of a human leukocyte antigen (*HLA*) class I molecule (*HLA-B27*) results in an abnormal host response to organisms such as YE causing reactive arthritis [20]. It is well documented that the prevalence of *HLA-B27* varies between populations worldwide [21]. There is a paucity of prevalence data regarding *HLA-B27* in the NZ population. One NZ study investigated the prevalence of *HLA-B27* in the NZ population (Caucasians and Māori only) but the focus of the study was ankylosing spondylitis, a rare type of arthritis that affects the spine [22] that is not clearly linked to yersiniosis.

Erythema nodosum may be induced by YE and has been reported to account for over 20% of erythema cases in Poland [23]. In a case–control study, erythema nodosum was reported by 3% of yersiniosis cases, compared to 0.1% of controls [17]. *Yersinia* have been implicated in the causation of inflammatory bowel disease (IBD), including Crohn’s disease (CD) [24]. However, a study of tissues from CD cases and inflammatory and non-inflammatory controls found *Yersinia* at similar frequencies in all three groups [25].

A case–control study of cases with chronic gastrointestinal disorders following acute bacterial gastroenteritis found significantly elevated odds ratios amongst yersiniosis cases for irritable bowel syndrome (IBS), functional constipation and gastroesophageal reflux disease [15]. Onset of IBS was more rapid following *Yersinia* infections than for other triggering organisms. It has been suggested that YE may be involved in the pathogenesis of Grave’s disease, an autoimmune disease, involving pathology of the thyroid gland [23]. However, a prospective cohort study found no relationship between the development of autoimmune thyroid disease and positivity for YE antibodies [26].

Sequelae affecting the kidneys (glomerulonephritis) and the heart (myocarditis) have also been reported internationally [27]. YP has been implicated in the progression of Kawasaki disease (KD), a form of vasculitis of unknown etiology. KD cases who were positive for YP antibodies were more likely to encounter cardiac sequelae [28]. A Canadian study reviewed evidence for the association of various sequelae with certain microbial infections [29]. For *Yersinia*, acute kidney injury, erythema nodosum and reactive arthritis were considered to be established sequelae, while Grave’s disease, and IBS were considered to be potential sequelae. *Yersinia* spp. infections were most frequently associated with reactive arthritis, followed by IBS, other joint outcomes and CD [30].

## 3. Has the Introduction of Culture-Independent Diagnostics Testing Influenced the Increase in Notified Cases?

A 2009 survey of NZ diagnostic laboratories showed that 34/35 microbiology laboratories across NZ were routinely testing all diagnostic fecal samples for *Yersinia* spp. by culturing to a selective medium [31]. Since this time, laboratories have merged and consolidated across the country and 14 laboratories are now screening fecal specimens, and all are routinely testing for *Yersinia* [32].

**Figure 4:**
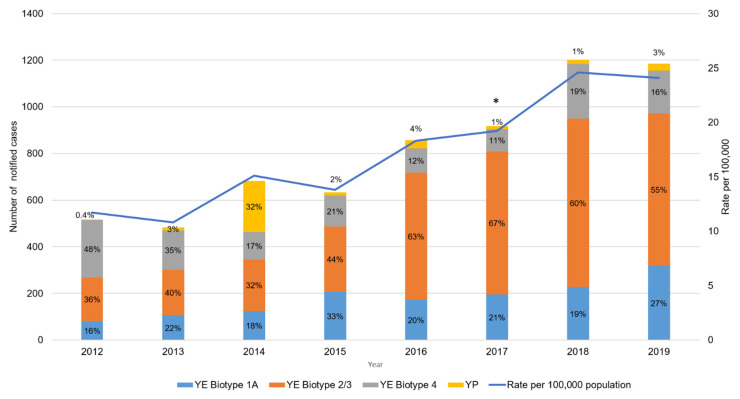
Total number of notified human yersiniosis cases in New Zealand between 2012 and 2019 as recorded in EpiSurv. Distribution of cases per annum (and percentage per year) represented according to *Yersinia enterocolitica* (YE) biotype (1A, 2/3 and 4) or *Yersinia pseudotuberculosis* (YP). Rate is calculated as the number of cases per 100,000 population, based on the Statistics New Zealand mid-year population estimates. * Indicates when the first major diagnostic laboratory introduced a culture-independent diagnostic test that included *Yersinia enterocolitica* (June 2017).

From 2015, NZ diagnostic laboratories have progressively introduced culture-independent diagnostic testing (CIDT) for fecal samples in order to improve diagnosis and enable organism-specific intervention. The first fecal CIDT that included *Yersinia* was not introduced in NZ until June 2017 (Figure 4, Section 4). Therefore, the increase in yersiniosis cases reported in NZ from 2010 to 2017 is not due to the introduction of CIDT testing.

Currently, 80% of all fecal samples nationwide are being tested via CIDT across commercial platforms including EntericBio^®^Dx (Serosep, West Sussex, UK), BDMax™ (Becton Dickinson and Company, Franklin Lakes, NJ, USA), Biofire^®^FilmArray^®^ (bioMérieux, Marcy-l’Étoile, France) and Ausdiagnostics (Sydney, Australia). NZ clinical diagnostic laboratories are accredited to the international laboratory quality standard ISO 15189 [33], which requires all new and alternative methods to be adequately verified before being brought into routine diagnostic use. *Salmonella* and *Campylobacter* notification rates have remained stable over the five years since the introduction of CIDT, suggesting that CIDT has had minimal impact on the rate of detection of these particular pathogens. This suggests that the same would apply for YE but further work is required to confirm.

CIDT has had a negative impact on YP case recognition as 50% of all NZ fecal samples are now being tested by panels that do not detect YP (EntericBio^®^Dx, BDMax™ and Biofire^®^FilmArray). This is of concern as it affects NZ’s ability to readily detect YP outbreaks such as that seen in 2014 [5]. While CIDT may improve the timeliness of pathogen detection, the absence of an isolate for subsequent typing can negatively impact on public health surveillance and outbreak detection. In addition, it may be difficult to test for and thereby monitor antimicrobial resistance. Since laboratory testing methods significantly affect both diagnosis and public health surveillance, any potential changes should be planned in consultation with stakeholders such as clinicians, public health professionals, epidemiologists and health planners [34]. Diagnostic laboratories in NZ are requested to reflex culture all *Yersinia* CIDT-positive samples and forward isolates to ERL for typing. In general, laboratories are compliant, with typically >90% of case isolates being referred.

## 4. The Types of *Yersinia enterocolitica* Causing Human Yersiniosis Have Changed in New Zealand

Pathogenic *Yersinia* have been historically defined as those harboring a 70 kb virulence plasmid (pYV), which has genes encoding adhesin A (*YadA*), various *Yersinia* outer proteins (Yops) and a transcriptional regulator gene (*virF*); as well as chromosomal genes invasin (*inv*), attachment and invasion locus (*ail*), *Yersinia* stable toxin A (*ystA*), and mucoid *Yersinia* factor A (*myfA*) [35]. In addition, a subset of these strains harbor high-pathogenicity islands (HPIs), which confer the capacity to cause disseminated infection [36].

There are six internationally recognized YE biotypes (BTs) (1A, 1B, 2, 3, 4 and 5) [37]. These BTs are based on a biochemical scheme but interpreting biotyping reactions can be subjective and misidentification of YE BTs is common [38]. Before 2017, the NZ Enteric Reference Laboratory (ERL) used biotyping as the primary epidemiological typing method and YE BT4 was the historically predominant BT in the years up to and including 2012. An increase in the proportion of notified cases identified as YE BTs 2 and 1A and a decrease in the proportion of notified cases identified as YE BT4 have been observed since 2013 (Figure 4). A NZ study showed that YE BT2 and BT3 clustered together using multiple locus variable-number tandem repeat analysis (MLVA) and core single-nucleotide polymorphism (SNP) analysis [39]. Reuter and colleagues [40] also observed the same finding using whole-genome MLST (wgMLST). As a result, since 2018, the ERL classifies these two BTs collectively as YE BT2/3.

YE BTs can also be subdivided into numerous (>48) serotypes based on their heat-stable somatic antigens [41]. The NZ ERL introduced serotyping in 2017 to extend typing within the YE BT2 group. Serotyping together with BT provides a combined phenotype (bioserotype). The combined bioserotype assists in narrowing epidemiological investigation by excluding confounding cases of the same BT, but different serotypes. The NZ emergent strain is bioserotype 2/3 O:9. For the years 2018 and 2019, BT2/3 O:5, 27 accounted for just 5% of all NZ BT2/3, and a single BT2/3 O:3 was confirmed. Bioserotypes 1B/O:8 (rarely seen in NZ), 2/O:5, 27; 2/O:9; 3/O:3; and 4/O:3 have been associated with human infection in various countries. Prior to 1990, YE serotype O:9 had not been isolated in NZ, despite the recovery of a number of other YE bioserotypes from humans and animals. However, since 1990 YE serotype O:9 has been isolated from human fecal samples with increasing frequency [42,43]. Discussion at a recent Australian NZ Enteric Reference Network meeting (November 2020) confirmed that *Yersinia* isolation rates across Australia are low and that the emergent NZ type YE 2/3, O:9 is rarely observed [44].

Unlike many other countries, YE BT1A is notifiable in NZ. Between 2012 and 2019, 1150 out of 4896 clinical YE isolates (23%) were identified as YE BT1A (Figure 4). This biotype, which includes a wide range of serotypes, has been considered as non-pathogenic internationally because it lacks the classical YE virulence determinants [45,46]. However, growing epidemiological and experimental evidence suggest that YE BT1A can cause gastrointestinal disease [47], and recent studies suggest that YE BT1A may trigger reactive arthritis [48]. Isolates of YE which lack the well-recognized virulence factors may cause disease through an alternative mechanism [49]. The *ystB* gene, which encodes the heat-stable enterotoxin Y-STb, may be part of this alternative mechanism and contribute to pathogenesis [50]. However, there is no current evidence to suggest that severity/occurrence of disease is linked to the presence of *ystB* gene. Preliminary analysis of a selection of NZ YE BT1A clinical isolates has shown that the majority (91/93) possessed the *ystB* gene (unpublished data). Other studies have also reported this observation [47,51].

YE BT 1A currently accounts for >20% of NZ *Yersinia* cases and work is ongoing regarding pathogenicity, diversity within the type, and sources for human case isolates.

## 5. Whole-Genome Sequencing Is Providing Unprecedented Discriminatory Typing Power

Biotyping and serotyping together do not provide enough discrimination to link bacterial isolates related to the same source while excluding non-related isolates as required to enable effective disease surveillance and outbreak investigations [52]. In a previous NZ study, Pulsed-Field Gel Electrophoresis (PFGE), using restriction enzymes *Apa*I and *Not*I, was shown to have sufficient discriminatory power to type YE BT1A, but not YE BTs 2, 3 or 4 [53].

From 2010, a MLVA assay combining previously described loci [54,55] was applied in NZ. This tool was used to type isolates from an outbreak of YE BT2/3 O:9 in 2016 attributed to a food premises serving sushi [34,39]. Of the 20 isolates available for MLVA typing, 19 cases had the same MLVA profile and one had a very similar profile, thus MLVA typing supported epidemiological evidence that isolates may be attributed to a point source. However, allelic variation between epidemiologically linked MLVA profiles did occur, suggesting that MLVA may be over-discriminatory and unlikely to provide useful information for long-term routine surveillance.

WGS has revolutionized the ability to compare and characterize foodborne pathogens and has accelerated the identification of foodborne outbreaks, allowing clustering of as few as two cases [56]. WGS offers unprecedented resolution for the characterization of YE [57]. The rapidly declining cost of WGS allows application in food safety management as well as real-time surveillance of foodborne disease and outbreak investigations. As a result, international researchers continue to fine-tune the criteria required to ensure robust and reproducible comparisons [58].

There are two main analytical approaches to analyze WGS data and understand the genetic relationships between isolates of interest. Single-nucleotide polymorphism (SNP) analysis assesses a WGS output base by base, while a MLST approach involves a gene by gene analysis that can include core genes (cgMLST) [56]. The use of WGS data and these analytical approaches has enabled scientists to establish the phylogenetic and population structure for the *Yersinia* genus [38,40,59]. Given the heterogeneous pathogenic potential of *Yersinia* members, identification to species and infra-species levels is essential for case confirmation and notification. For example, YE BT1A are genetically the most heterogeneous of all the YE BTs and it has been proposed that this BT may represent more than one subspecies [46]. Recently, seven putative novel species of *Yersinia* were identified by Savin and colleagues using an automated taxonomic assignment procedure with species-specific thresholds based on a core-genome multilocus sequence typing (cgMLST) tool [45]. Two of those species, *Y. artesiana* and *Y. proxima*, have been isolated from human stool samples and were initially identified as YE BT1A [46]. These two species have not yet been confirmed in NZ.

One phylogenetic study on the entire *Yersinia* genus identified a phylogenetic split for YE on the basis of high pathogenic (BT 1B), low pathogenic (BT2-5) and non-pathogenic (BT1A) [40]. The same study used core-genome SNP analysis to show that phylogenetic separation of clinical isolates belonging to BT2 and BT3 of YE was concordant with serotype and not BT. This is due to the difficulties in interpreting variable reactions discriminating BTs 2 and 3 for the traditional biotyping methods [38,40]. In addition, phylogenetic analysis of WGS data has also revealed a high variability among YE genomes as an entire species but a lower diversity in the BT2-5 groups, particularly for the serotypes O:5 27, O:3 and O:9 [40,60,61].

Results from a comprehensive genome-scale analysis of YP incorporating isolates attributed to the 2014 NZ outbreak and international data indicate that these NZ strains represent a geographically isolated clade of YP [5]. A recent study analyzed clustered regularly interspaced short palindromic repeat (CRISPR) loci within 134 YP isolates from 19 different countries collected over a 46 year time frame from a wide host range and a full spectrum of serotypes [59]. This included four isolates from animals in NZ. The study reported that YP had a clear phylogeographic split in its population, with an Asian ancestry and subsequent dispersal of clonal lineages into Europe and the rest of the world, including NZ.

A genus-wide seven-gene MLST scheme (known as the McNally scheme) was developed by Hall and colleagues [38] which allowed both the identification of *Yersinia* species and the differentiation of *Y. enterocolitica* biotypes. The improvement of whole-genome sequencing has allowed for the scheme to be extended to incorporate cgMLST genotyping [60], which provides much improved resolution and phylogenetic precision.

The purpose of the cgMLST scheme of Savin and colleagues [60] is species-level and bioserotype-level identification. Whether this scheme could also be useful as a tool in epidemiological investigations of *Yersinia* outbreaks is not yet clear. EnteroBase also incorporates cgMLST and wgMLST schemes for *Yersinia* and incorporates a hierarchical clustering method of cgMLST sequence types to allow mapping of bacterial strains to predefined population structures [61]. Distances between genomes are calculated using the number of shared cgMLST alleles and genomes are linked on a single-linkage clustering criterion. These clusters are assigned a stable cluster group number at different fixed cgMLST allele distances [61,62]. However, to date, the application of the EnteroBase scheme for public health surveillance or outbreak investigation has not been observed in the literature and is also not currently used in NZ for these purposes.

WGS analysis of human clinical isolates in NZ is currently only performed if an outbreak is suspected. As a result, current WGS typing of clinical *Yersinia* in NZ has occurred ad hoc or as a part of an outbreak investigation prospectively or retrospectively [5,39]. Currently, ESR performs WGS on the Illumina NextSeq platform using the Nextera XT library kit (Illumina, San Diego, CA, USA). Sequencing quality assessment, species identification, *de novo* assembly, and sequence type assignment are performed using Nullarbor v 2.0. [63]; and an in-house pipeline infers ST using the McNally scheme [38] and Achtman schemes (used for YP only) [64]. Genetic relationships between *Yersinia* isolates are assessed using two core-SNP analysis methods: Snippy 4 [65] and SnapperDB [66]. Both methods map sequence reads against a reference genome. SnapperDB also provides hierarchical typing information, including a SNP address (a numerical code describing the population structure at seven different SNP thresholds) [66].

To date, ESR has sequenced >400 *Yersinia* isolates including 25 isolates from pork sources within NZ. The dataset is disproportionately skewed as WGS is also being used to assist in the identification of biochemically atypical strains. SNP analysis demonstrates that YE clusters according to bioserotype, an observation that is consistent with other phylogenetic studies [38,40]. Using WGS data to infer ST, it has been observed that ST12, ST14 and ST18 correlate to bioserotypes BT2/3, O:9; BT2/3, O:5, 27; and BT4, O:3, respectively. However, ST has limited discrimination for outbreak investigations [39]. Core-SNP analysis offers a high level of resolution and supported epidemiological evidence for a YE BT2/3, O:9 (ST12) outbreak that occurred in 2016, demonstrating epidemiologically linked cases clustered together with less than five SNP differences between them, suggesting a potential common point source [39]. Similar observations were made for the YP outbreak, where the ST42 subclade comprising the outbreak isolates (*n* = 82) had a maximum SNP distance of two SNPs to the reference genome used, again suggesting a potential common source [5].

**Figure 5:**
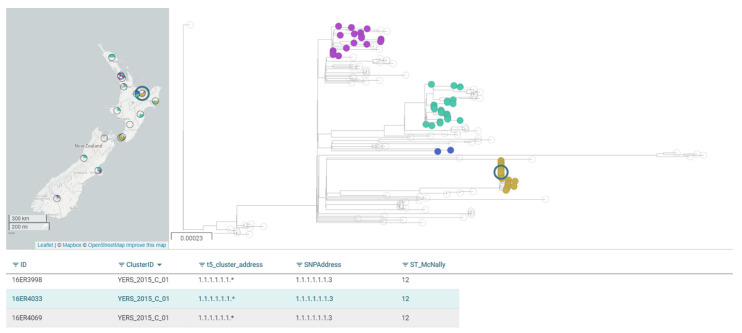
Microreact visualization of the 164 *Yersinia enterocolitica* biotype 2/3, O:9 ST12 New Zealand isolates whole-genome sequenced to date. The three isolates shown in the table pertain to a 2016 outbreak. These isolates are also highlighted within the khaki-colored cluster in the phylogenetic tree. The tree is a maximum likelihood tree using core single-nucleotide polymorphic (SNP) differences identified using Snippy 4 [65]. IQ-TREE [68] was used for tree construction with 2000 ultrafast bootstrap [69]. Scale shown within the tree window indicates nucleotide substitutions per site.

ESR is currently using a locally derived YE ST12 reference genome for core-SNP analysis to cluster isolates of the emergent YE BT2/3, O:9 ST12, and the Microreact Open Data Visualization and Sharing for Genomic Epidemiology tool to visualize and further analyze clustering data [67]. Figure 5 shows a snapshot of this visualization for the emergent strain. Specifically depicted in this figure is a previously described outbreak ([34,39]). The tree represents Snippy analysis and the table represents SnapperDB-derived information. Data show that the 164 YE BT2/3, O:9 ST12 isolates sequenced to date exhibit limited diversity as all are within 100 SNPs. Currently, SnapperDB is the primary clustering tool with a cut off of 5 SNPs and Snippy 4 is being used in parallel for validation purposes. No inconsistencies have been noted between the two methods within the current dataset.

## 6. Key Reservoirs, Sources and Transmission Routes of *Yersinia* in New Zealand Require Further Exploration

### 6.1. Foodborne Transmission

Based on epidemiological information, the majority of NZ human yersiniosis cases are considered sporadic and without an identifiable source [70]. Notified outbreaks of yersiniosis in NZ are often small so the information from these is limited [70]. Data collected from outbreak investigations, and historical NZ studies including a case–control study performed in 1995–1996, identified risk factors that include consumption of pork products, association with backyard slaughter of pigs (possible zoonotic exposure), animal contact, possible person-to-person contact, and contact with untreated water or unreticulated sewage [70,71,72]. In 2016, an outbreak of YE BT2/3 serotype O:9 involving 24 cases was reported in the Bay of Plenty region in NZ. A source for this outbreak was not confirmed but MLVA typing showed a common outbreak profile among cases who had eaten from sushi premises supplied by the same kitchen. Probable sources included an infected food handler(s), contaminated ingredients at the implicated premises and/or dispersed food ingredients [34]. Mandatory screening of food handlers is not undertaken in NZ but notified cases at risk of exposing others, for example, food handlers, health care or childcare workers are required to stand down until symptom free for 48 h [73]. The rate of carriage of *Yersinia* in healthy individuals is currently unknown.

There are no published source attribution studies for *Yersinia* for NZ or internationally. However, there are published expert elicitations studies which include YE for the USA, Canada and England and Wales and NZ [74,75,76,77,78]. A NZ expert elicitation study reported that food was estimated (63%, 29–91%, 95% percentile credible interval) as the primary route of transmission for YE infections [74]. This estimate is lower compared to estimates from other countries, but it is uncertain whether these differences represent true differences in disease etiology or differences in the opinions of the expert groups used in the various studies [74].

The sources of yersiniosis caused by YP are less clear. The NZ YP outbreak in 2014, involving 220 cases, represents one of the largest ever reported outbreaks of YP [5]. Disease outbreaks that involve YP are rare in other countries and no outbreaks had been previously reported in NZ. Investigations into the NZ outbreak did not identify a confirmed source, but consumption of fresh produce, specifically lettuce or carrots, was reported to be a risk factor [79]. Recent outbreaks caused by YP internationally have been associated with fresh produce (Table 1). As highlighted in the YP outbreak investigation report, there are several knowledge gaps around *Yersinia* in NZ, in particular the baseline prevalence of these pathogens in foods and the environment is unknown and thus the epidemiology of *Yersinia* remains unclear. Published data on the prevalence of *Yersinia* on fresh produce in other countries are also scarce and historical.

International studies have reported that pigs are an important reservoir for YE and the pathogenic YE bioserotypes that are most frequently found in pigs and pork products are those most commonly reported in human infections [6]. Often YE infections are linked to the consumption of undercooked contaminated pork or cross-contamination of other food items during handling and preparation of raw pork [93,94,95]. Several case–control studies (Table 2) and a systematic review and meta-analysis study [96] published internationally support this observation. However, other food items such as raw milk, pasteurized milk, water, fresh vegetables and produce have been implicated, suggesting that sources other than pork may also be important [70,71,82,96,97,98].

Few data on the prevalence of *Yersinia* in NZ pigs and pork are available in published literature and are historic. *Yersinia* can be carried in pigs without symptoms and this animal group is therefore not represented in studies based on testing of sick animals in NZ veterinary diagnostic laboratories [43]. A study in the 1990s, tested 200 pigs (tonsils) over a 12 month period for *Yersinia.* During the same period, 70 retail diced or ground pork samples were purchased from supermarkets and tested for *Yersinia.* Of the 200 pig samples, 28.5% (*n* = 57) and 31% (*n* = 62) were positive for YE and YP, respectively. YE bioserotypes BT4, O:3; BT2, O:5, 27 and BT1A were identified amongst the isolates recovered. Of the 70 retail pork products tested, 27.1% (*n* = 26) were positive for *Yersinia*. However, only three samples were confirmed to contain YE BT 3 (serotype not confirmed) and 11 samples contained YE BT1A. The remaining samples contained other *Yersinia* species that are not recognized as pathogenic [103]. A small microbiological survey of ready-to-eat pork products performed in the early 1990s detected YE in 2 out of 34 samples but only YE BT1A was isolated [104].

A number of international published studies have reported the isolation of YE from pigs on farm and during slaughter (Table 3 and as reviewed by [105]) as well as in pork products [3,70,106,107,108,109]. The prevalence of YE across studies is variable and can depend on several factors including age of the animals tested, farm management and biosecurity levels (as reviewed by [105]), and most importantly the sampling and detection methodology used. It is difficult to achieve a multivalent isolation method for all *Yersinia* or only for pathogenic YE and YE [3,110], so it is likely that differences in results are, in part, due to the methods used. This is also the case the with NZ studies outline above where slightly different methodologies were used between studies [103,104].

*Yersinia* is present in the oral cavity, especially the tonsils, submaxillary lymph nodes, and the intestine and feces of pigs. Cross-contamination to the carcass as a result of spread of the organism via feces, intestinal contents and tonsils can occur during slaughter and dressing operations [105]. One cross-sectional study reported that the initial presence of YE in the tonsils and/or feces was significantly associated with carcass contamination at all sampled areas. Other risk factors for carcass contamination identified in that study included splitting of the head together with the carcass, and incision of the tonsils during removal of the pluck [111]. Blast chilling of carcasses for 1 h was reported to not significantly affect the occurrence of YE on the carcasses [112]. Mitigation strategies within slaughter practices (e.g., putting bags over pigs’ heads to prevent YE from pharynx contaminating food), and storage or processing activities (e.g., surface sterilization, additional product testing) have been reported to be effective internationally [106,107]. Modelling consumer practices and scenarios demonstrated that the number of pork mince packages containing high numbers of pathogenic YE are expected to cause the highest risk of yersiniosis and is primarily influenced by consumer storage practices. A reduced storage time (under one day) or a storage temperature (below 4 °C) would largely reduce the proportion of packages containing high numbers of pathogenic YE [113].

### 6.2. Possible Transmission Routes from Other Animal Species

International literature reports that other healthy animal species besides domestic pigs can harbor YE and YP, including domestic and wild animals (e.g., wild boar, deer, rodents and birds) [125,126,127,128,129]. Some of these wild animals such as wild boar and deer are hunted and farmed for consumption in some countries, including in NZ, thus presenting a risk of transmission of foodborne disease [125,130]

Wild animals may also serve as natural reservoirs of YE and contribute to the environmental circulation of the bacterium. However, epidemiological links between wild, farm or domestic animals and human yersiniosis are yet to be established [125,131]. A Finnish study compared YE and YP isolates from wild boar and domestic pigs and suggested that wild boars and domestic pigs may serve as reservoirs for different YE and YP strains [131]. The role of these animals in the epidemiology of *Yersinia* in the environment is unclear, especially as outbreaks attributed to fresh produce may point to contamination events occurring through contaminated irrigation water, fertilizer or other environmental sources [80]. A study investigating the occurrence of YP in iceberg lettuce and the environment in Finland suggested that wild animals may access lettuce fields and could contaminate water sources, soils and the lettuces with their feces [132].

Little data exist in the published literature on the prevalence of *Yersinia* in healthy animals in NZ are available in the published literature. One NZ molecular study screened fecal samples from healthy, farmed red deer at slaughter and reported an overall prevalence range of YE and YP on a per animal basis of 2.43 to 11.17% and 0.49 to 2.91%, respectively [133]. However, isolation of YE or YP was not attempted. Earlier studies in the 1990s reported isolating YE serotype O:9 from deer and cattle which had shown non-specific reactions in serological testing for brucellosis [42,43].

Between 1988 and 1996, 347 isolates of YE and YP from animals were obtained from veterinary diagnostic laboratories around New Zealand for confirmation of identity, biotyping, serotyping and virulence testing [43]. While many of the isolates were from clinical (sick animal) cases, a few originated from healthy animals surveyed in response to outbreaks of disease or for other purposes, such as *Brucella abortus* surveillance.

Enteropathogenic bioserotypes of *Yersinia* including BT2, O:9; BT2 O:5, 27; and BT4, O:3 were isolated from various animal species (Table 4). YE BT4, O:3 was not identified among the isolates from pigs in this survey, which is in contrast with results obtained from a survey undertaken from pigs during processing [103], thus supporting the asymptomatic nature of porcine infections. The lack of isolation of YE bioserotype 2, O:9 from pigs during this time, led to the suggestion that cattle were the principle reservoir for human infection in NZ, with a range of other domestic animals acting as secondary sources [43]. YE BT1A accounted for 12% (42 out of 347) of the isolates tested and were predominately from deer and cattle, but were also recovered from other animal species. Studies from other countries have reported that YE BT1A is commonly isolated from animals but most often regarded as non-pathogenic [125,131].

Yersiniosis attributed to direct contact with farm animals is rarely reported in the literature. A NZ study associated the handling of cattle (relative risk = 4.88; *p* = 0.008) and sheep (relative risk = 14.80; *p* = 0.001) with an increased risk of infection [72]. This risk factor was identified in a predominantly rural region of NZ [70]. A systematic review and meta-analysis of risk factors of YE using data from international case–control studies reported that occupational exposure to pigs was significantly associated with sporadic YE infections [96]. Domestic animals have also been suspected as being sources of human yersiniosis because of their close contact with humans, especially young children [43,134]. However, transmission from pets to humans has not yet been proven. Pathogenic YE may be transmitted to humans indirectly from pork and offal via dogs and cats [135]. The frequency of contacts between various animal sources and humans could reflect the risk of infection caused by the different groups of pathogenic *Yersinia* [136]. Well animals are more likely to enter the commercial food chain and cause foodborne infection. Sick animals are more likely to cause zoonotic infection via direct or indirect contact with the animal or their body products. Sick animals should not be entering the NZ commercial food chain, but may contribute to foodborne illness via indirect pathways not yet understood.

### 6.3. Waterborne Transmission

Outbreaks of YE have been attributed to the consumption of untreated drinking water internationally [137,138]. A one-year study of gastrointestinal disease in a predominately rural region of NZ during the period 1993–1994 reported that consumption of water from a home supply had a statistically significant threefold increase in the risk for intestinal YE infection. This risk factor may be different for urban populations [70]. In NZ, rural households may draw their drinking water from surface, ground, or rainwater sources which are not subject to the same drinking water standards as community water supplies [139] and may be contaminated by animals or birds. Studies internationally have reported the detection of pathogenic YE in environmental waters [140] and untreated water (and sewage) [141]. Published *Yersinia* prevalence data for NZ water sources are not available.

### 6.4. Human-to-Human Transmission

Yersiniosis is rarely transmitted through sustained person-to-person transmission, but there have been previous YE outbreaks internationally in which a food handler was implicated [142,143]. A nosocomial outbreak of diarrheal disease due to YE has been reported in Canada [144].

Asymptomatic bacteremia in blood donors has historically led to fatal transfusion outcomes in NZ. In 1997, it was reported that eight cases of transfusion-associated transmission resulting in five deaths had occurred in NZ in the preceding five years [145]. The New Zealand Blood Service has implemented several intervention measures since this time such as deferrals for donors for three months from recovery post *Yersinia* infection, (and *Shigella, Salmonella* or *Campylobacter*); deferral of contacts with infected individuals for four weeks from last contact; leucoreduction in all blood components; and performing visual checks on blood products at a number of stages through processing and issue [146]. These interventions appear to have dramatically reduced transfusion-associated transmission of YE as no cases were recorded between 2011 and 2019 [147], despite the national rate of yersiniosis increasing over this time.

## 7. Yersiniosis Is Also Increasing in Animals in New Zealand

**Figure 6:**
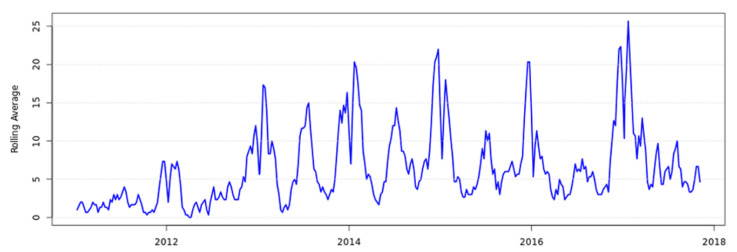
Bovine *Yersinia* notifications in New Zealand (3 week rolling average), 1 January 2011–31 October 2017. Data provided by New Zealand Ministry for Primary Industries.

Samples from sick animals in NZ are tested at veterinary diagnostic laboratories. Specific culture for *Yersinia* is performed following veterinarian request using Cefsulodin Irgasan™ Novibiocin (CIN) agar. *Yersinia* are routinely identified to species level, but further typing is not routinely performed. NZ national veterinary diagnostic data are collated by the NZ Ministry for Primary Industries. Based on NZ national veterinary diagnostic data available in 2017, it appears that animal yersiniosis was increasing. Figure 6 represents the number of bovine yersiniosis notifications in NZ between 1 January 2011 and 31 October 2017 [148].

A small, previously unpublished NZ pilot study typed a selection of *Yersinia* isolated from sick animals by diagnostic veterinary laboratories between January and March 2018. The results for the 93 isolates are shown in Table 5.

These isolates were analyzed using whole-genome sequencing (WGS) as described in Section 5. Most isolates were confirmed as YP ST19 (Achtman scheme). All YP ST19 isolates in this study harbored the *ail* and *inv* virulence genes and most were positive for *yadA*. A representative subset of 10 of these isolates were tested for melibiose and all were found to be negative for fermentation of this carbohydrate. It has been claimed that melibiose-negative YP O:3 strains of genetic group G5 (ST19) are associated with lowered pathogenicity in humans [64] and are also carried by healthy pigs [149,150]. Melibiose-negative YP ST19 strains have been reported to cause severe, sometimes fatal, diarrhea in cattle, abortions in cattle and sheep and fatal enteric disease in squirrel monkeys [151,152,153]. These strains have also been isolated from humans with enteric symptoms both in NZ and elsewhere [5,150,154]. The frequency of melibiose-negative YP ST19 in NZ cattle and the infrequent isolation of YP in general in NZ human clinical cases suggest that the NZ melibiose-negative YP ST19 is less pathogenic for humans than cattle.

Two cattle isolates from the same study were identified as YE BT5, ST13 and carried the virulence genes *ail*, *inv*, *yadA*, *ystA* and *virF*. These two strains were confirmed as serogroup O:3 (O:2 antiserum was not available for testing). From a background dataset of >7000 clinical isolates, BT5 has only been reported in a single case of NZ human infection that occurred in 2003. A 2020 search of EnteroBase *Yersinia* database [155] showed only 12 ST13 strains with a mix of human and animal sources. Given the high historic prevalence in NZ animals [43] and the paucity of detection in NZ humans, BT5 O:2, 3 ST13 may hold key information as to what genes are necessary for *Yersinia* to cause clinical illness in humans but are absent from this strain.

The single bovine isolate of YE BT2/3 O:9 ST12 isolated in this study clustered within 25 single-nucleotide polymorphism (SNPs) of 164 NZ YE BT2/3 O:9 ST12 clinical isolates (Section 5).

This 2018 study was based on a small sample size and covered a short time frame. The study population was dominated by cattle rather than representing the range of animal species of an earlier NZ study [43]. However, results indicate that the current increase in yersiniosis in NZ cattle appears unrelated to the current increase in NZ clinical cases, but cattle can harbor strains genetically similar to the emergent human YE BT2/3 O:9 strain.

It is recommended that a more detailed study including a greater range of animals over a longer time period now be undertaken.

## 8. Isolating *Yersinia* from Foods Can Be Challenging

In NZ, routine food surveillance for *Yersinia* is not performed. One of the difficulties in gathering robust data lies in the isolation of *Yersinia* from foods using traditional culture methodology. Microbiological culture methods remain the gold standard for the detection of pathogens from naturally contaminated sources including food, water, environmental and clinical samples. Various standard culture-based methods have been described for the detection and isolation of YE and YP from food samples [3,156,157].

The source of *Yersinia* can markedly affect probability of isolating the organism from culture. The low numbers of pathogenic *Yersinia* usually present and the high background microbial population (which is capable of growing more rapidly than pathogenic *Yersinia*) hampers detection and isolation methods. As a result, direct isolation of pathogenic *Yersinia*, even on selective media is seldom successful. This problem can be partly addressed by including an enrichment step before the use of selective media. Several enrichment methods have been suggested for the recovery of YE in foods [3]. Unlike other enteric bacteria, YE is psychrotrophic and therefore “cold” (10 °C) enrichment is commonly used. However, the long incubation periods required for adequate *Yersinia* growth also allows the growth of other psychrotrophic bacteria, limiting its effectiveness. To decrease competing background flora, alkali treatment is also used for the enrichment of YE, as it can tolerate alkaline conditions, in contrast to other Gram-negative bacteria [3].

The International Organization for Standardization (ISO) method for YE from foods (ISO 10273) [158] was revised in 2017 to include direct plating on CIN agar, and shortened incubation times for Peptone Sorbitol Soy (PSB) broth (from 5–6 days to 44 ± 4 h) and plating from the enrichment onto CIN agar 24 ± 2 h rather than 28 h, both of which had been previously recommended [159,160,161]. An additional selective enrichment broth, Irgasan™, ticarcillin and potassium chlorate (ITC) which was specifically designed for the improved isolation of YE serogroup O:3 from meat products [162], is also recommended within the ISO method. The validation study reported limits of detection (LOD) of 9.4 colony forming units (cfu)/25 mL raw milk, 9.9 cfu/25 g minced meat and 63 cfu/25 g lettuce samples and recovery of pathogenic YE on CIN was most efficient after KOH treatment [161]. This ISO method (using both PSB and ITC) has been implemented in NZ for microbiological surveys of foods.

The use of CIN agar is considered the best medium for YE isolation and its use is recommended within the ISO methods and Food and Drug Association-Bacteriological Analytical Manual (FDA-BAM) [163] method. However, limitations of this media have been identified including (a) inhibition of some YE 3/O:3 strains, (b) a number of *Enterobacterales species* may grow on it and produce colonies quite similar in appearance to YE (leading to misdiagnosis if a limited number of presumptive colonies is picked for identification), (c) it lacks the ability to differentiate between potentially virulent YE and non-pathogenic strains or other *Yersinia* spp., and (d) the growth of YP may also be inhibited on this media [3,164]. The method is also time consuming as many non*-Yersinia* colonies need to be further identified and excluded using biochemical tests and PCR detection. Several molecular methods, in particular PCR and RT-PCR assays, have been described for the detection of pathogenic YE or YP in food. These assays often employ the detection of one or more virulence-related targets including chromosomal genes such as *ail, inv* or *yst*, or the virulence plasmid-borne *virF* gene [3,165,166,167,168,169,170,171,172,173]. Studies have indicated a higher prevalence of pathogenic YE using PCR than culturing [3].

The ISO method (ISO 18867:2015) specifies real-time polymerase chain reaction (RT-PCR) assays for the detection of pathogenic bioserotypes of YE and for YP [174]. DNA-based methods, including RT-PCR, offer the advantage of detecting a pathogen more rapidly and with greater sensitivity compared to culturing methods [3]. However, YE and YP are normally present in foods in low numbers which are below levels that are directly detectable by the RT-PCR meaning enrichment of the food is required to increase the numbers of *Yersinia* prior to detection by RT-PCR [110]. The use of a concentration step such as immunomagnetic-separation as used for other foodborne bacterial pathogens could potentially increase the numbers of *Yersinia* available for detection and also alleviate the non-specific amplification and inhibition that is currently observed. Many molecular methods including the ISO method, use the *ail* gene as the primary gene target meaning they do not permit the detection of YE BT1A strains, which often lack this gene. As the status of this particular BT in human infection in NZ is undetermined, improving detection methods for this BT is essential for understanding the epidemiology of this BT in NZ. The use of a combination of gene targets such as *ystA*/*ystB* [125,175] or *foxA* [176] alongside *ail* may be appropriate to detect both the enteropathogenic YE and YE BT1A.

## 9. Antimicrobial Susceptibly Data for *Yersinia* in New Zealand Is Currently Limited

In NZ, diagnostic laboratories do not routinely perform antimicrobial susceptibility testing on fecal *Yersinia* isolates [32] and a national *Yersinia* AMR microbiological survey is yet to be undertaken, thus there is a paucity of AMR data on NZ isolates. Overseas studies have reported that YE are intrinsically resistant to clinically important antibiotics, such as amoxicillin, amoxicillin-clavulanic acid and first-generation cephalosporins, due to the production of one or two chromosomally encoded β-lactamases, encoded by the *blaA* and *blaB* genes [175,177,178,179,180]. Studies have reported that YP is susceptible to most clinically important antibiotics, but multidrug-resistant strains and colistin-resistant phenotypes have been observed [128,181,182,183,184]. It has been reported that with the exception of outer membrane, no natural mechanisms of resistance exist for YP [128,185]. It has been suggested that the insensitivity to colistin may be attributed to mutations in genes responsible for the composition of lipopolysaccharide [128]. A comprehensive combined phenotypic and genotypic AMR study of NZ strains is planned.

## 10. Future Perspectives

Many domestic and farmed animal species have been shown to harbor pathogenic *Yersinia* either symptomatically or asymptomatically, but the transmission pathways from reservoir to human in NZ are unclear. Several knowledge gaps around *Yersinia* in NZ exist. There is a lack of baseline prevalence data of these pathogens in foods and the environment and existing data in NZ is historic. The significant increase in yersiniosis cases in NZ over the last 5–8 years highlights an urgent need to begin elucidating the epidemiology of *Yersinia* in NZ. International and NZ data indicate that yersiniosis is attributed to foodborne sources, in particular with YE and pork. However, a comprehensive case–control study and source attribution studies are required to identify the major risk factors and sources of yersiniosis in NZ. It may also be possible to ascertain whether there are particular exposures between ethnic groups that could be targeted with focused intervention strategies or education efforts. A ‘One-Health’ approach to collect and collate data from human, animal (both sick and healthy) and environmental sources in combination with WGS typing of isolates will help identify key transmission pathways where intervention strategies can be applied. The unprecedented power of WGS will not only improve future outbreak detections and public health surveillance but will also enable further in-depth genomic comparisons to be undertaken. A comprehensive analysis of NZ *Yersinia* genomes alongside international genomic data is required in order to better understand the phylogenetic and population structure of *Yersinia* in NZ in comparison with other countries. This type of genomic analysis will aid in the identification of endemic clones as well as providing insight into the evolution and appearance of new strains. A genome-wide association study incorporating a large number of pathogenic and non-pathogenic members of *Yersinia* will help elucidate which virulence genes may be playing a role in the clinical infection of cases of BT1A in NZ. This analysis may identify genes that are suitable as diagnostic markers to identify BT1A, which is currently not detected by food testing standard detection methods designed for enteropathogenic YE. In addition, prospective genomic comparison of YE BT5 ST13 from animal sources and human YE BT 2/3 and 4 will assist in determining key virulence genes absent from the apparently non-pathogenic strain.

In 2020, the NZ Health Research Council allocated funding for a three-year collaborative NZ project “Unravelling the mysteries of yersiniosis” and work will soon commence on addressing the knowledge gaps identified in this review [186].

## Figures and Tables

**Table 1:** Selected published outbreaks of *Yersinia* since 2000 attributed to food.

Country	Year	Number of Cases	Biotype/Serotype ^a^	Source	Reference
New Zealand	2016	24 (21 confirmed, 3 probable)	YE biotype 2	Not confirmed, suspected sushi	[34]
Sweden and Denmark	2019	57	YE 4, O:3	Spinach	[80]
Japan	2015	4	YE O:8	Not stated	[81]
Norway	2014	133	YE O:9	Mixed salad	[82]
Norway	2011	21	YE O:9	Mixed salad	[83]
United States of America	2011	16	YE	Pasteurized milk	[84]
Australia	2009	3	YE	Roast pork, Barbequed pork	[85]
Norway	2006	11	YE O:9	Processed pork	[86]
Japan	2004	16	YE O:8	Salad (containing apple, cucumbers, ham, potato, carrots and mayonnaise)	[87]
United States of America	2002–2003	9	YE O:3	Chitterlings (pig intestine)	[88]
New Zealand	2014	220	YP	Not confirmed, suspected produce	[5]
Finland	2014	55	YP O:1	Raw milk	[89]
Finland	2006	104	YP O:1	Raw carrots	[90]
Finland	2004	53	YP O:1	Raw carrots	[91]
Finland	2003	111	YP O:1	Raw carrots	[92]

^a^*Yersinia enterocolitica* (YE) or *Y. pseudotuberculosis* (YP) and biotype and/or serotype if reported.

**Table 2:** Selected published case–control studies involving *Yersinia enterocolitica* or *Y. pseudotuberculosis.*

Country	Year	Species (Outbreak) ^a^	Risk Factors	OR/aOR/mOR (95% CI)/*p* Value ^b^	Reference
New Zealand	1995–1996	YE	Consumption of pork	OR 1.34 (1.03–1.75)	[71]
			Eating food from a sandwich bar	OR 1.18 (1.09–1.27)	
Sweden	2004	YE	Eating food prepared from raw pork products	OR 3.0 (1.8–5.1)	[93]
			Eating treated sausage	OR 1.9 (1.1–3.3)	
			Use of a baby’s dummy	OR 1.9 (1.1–3.2)	
			Contact with domestic animals	OR 2.0 (1.2–3.4)	
Sweden	2019	YE (Outbreak)	Eaten spinach	aOR 1.4 (0.5–3.7)	[80]
Denmark			Eaten spinach	aOR 113 (3.7–3400)	
Norway	2014	YE O:9	Eaten salad	OR: 10.26 (0.85–123.57)	[82]
Finland	2006	YE (Bioserogroups 3–4/O:3, 2/O:9)	Eating or tasting raw or medium done pork	OR 6.6 (1.7–24.9)	[99]
			Eating in a canteen	OR 3.5 (1.6–7.9)	
			Eating in a restaurant	OR 6.1 (1.4–27.2)	
		YE Biotype 1A	Eating game meat	OR 0.5 (0.2–0.9)	
			Consumption of milk and milk products	OR 0.4 (0.1–1.0)	
			Consumption of imported fruits and berries	OR 3.5 (1.2–10.5)	
			Consumption of lettuce and cabbage	OR 0.3 (0.1–0.8)	
Germany	2009–2010	YE	Consumption of raw minced pork	aOR: 4.7 (3.5–6.3)	[100]
			Preparation of minced pork in the household	aOR: 1.4 (1.1–1.9)	
			Playing in a sandbox	aOR 1.7 (1.3–2.4)	
			Contact with birds	aOR 1.7 (1.1–2.6)	
Finland	1998	YP (Outbreak)	Consumption of iceberg lettuce	mOR: 3.8 (1.3–9.4)	[101]
Finland	2001	YP	Consumption of iceberg lettuce	mOR: 5.7 (1.6–47.7)	[102]
Finland	2014	YP (Outbreak)	Consumption of raw milk from a producer	mOR: 22.2 (3.6–∞)	[89]
			Raw milk in general	mOR: 16.9 (2.6–∞)	
Norway	1998–1990	YE	Consumption of pork items	*p* = 0.02	
			Consumption of sausage	*p* = 0.03	

^a^*Yersinia enterocolitica* (YE) or *Y. pseudotuberculosis* (YP). Some studies were associated within an outbreak as indicated. ^b^ Odds ratio (OR) with 95% confidence interval (CI). Studies reported adjusted odds ratio (aOR) which included adjustments for age and sex [80] or age, sex and region [100] alongside multivalent analysis, or matched OR (mOR) where controls were matched according to age, sex, and region [94,101] or where respondents that lived in the same household as the case but did not meet the case definition [89].

**Table 3:** Selected published studies reporting the prevalence of *Yersinia enterocolitica* (YE) or *Y. pseudotuberculosis* (YP) in pigs at or prior to slaughter since year 2000.

Country	Sample Tested	YE/YP	Prevalence (%)	Biotype (BT), Serotype	Reference
New Zealand	Tonsils	YE	57/200 (28.5%	BT4, O:3; 2, O:5, 27; BT1A	[103]
		YP	62/200 (31%)		
Belgium	Tonsils	YE	199/360 (55.3%)	Not reported	[111]
	Feces at slaughter		92/360 (25.6%)		
	Carcass		143/360 (39.7%)		
	Tonsils	YP	5/360 (1.4%		
	Feces at slaughter		2/360 (0.6%)		
	Carcass		1/360 (0.3%)		
Brazil	Carcass	YE	1/400 (0.3%)	BT4, O:3	[114]
	Tonsils		5/100 (5%)		
	Lymph nodes		2/90 (2%)		
China	Tonsils	YE	878/4495 (19.53%)	BT2, O:9; BT4, O:3; BT2, O:3; BT1A	[115]
	Intestinal contents		93/1239 (7.5%)		
	Feces		161/3039 (5.3%)		
	Tonsils	YP	4/4495 (0.08%)		
	Intestinal contents		0/1239		
	Feces		0/3039		
Finland	Tonsils	YE	234/388 (60.3%) PCR only	-	[116]
	Intestinal samples		94/356 (26.4%) By culture	BT4, O:3	
France	Tonsils	YE	414/3120 (13.7%)	BT3, 4, 5	[117]
Germany	Fecal during rearing period (final) fattening unit)	YE	96/491 (19.6%)	BT4, O:3	[118]
	Feces at slaughter		2/379 (0.5%)	BT4, O:3	
	Tonsils		143/372 (38.4%)	BT4, O:3; 2, O:9	
	Lymph nodes		13/346 (3.8%)	BT4, O:3	
	Carcass (before chilling)		1/393 (0.3%)	BT4, O:3	
	Carcass (after chilling)		0/383	BT4, O:3	
Italy	Fecal (cecal contents)	YE	77/451 (17.1%)	BT2, O:9; BT4, O:3; BT1A	[119]
	Tonsils		27/250 (10.8%)	BT4, O:3; BT1A	
	Carcass		11/451 (2.4%)	BT2, O:9; BT4, O:3; BT1A	
	Scalding water		4/34 (11.8%)	BT4, O:3; BT1A	
Italy	Tonsils	YE	55/201 (27.4%)	BT4, O:3	[120])
		YP	4/201 (2.0%)	Serotypes O:3, O:1	
Italy	Carcase swabs: finishing pigs	YE	0/126 (0%)	-	[121]
	Carcase swabs: piglets		0/35 (0%)		
	Colon contents: finishing pigs		15/126 (11.9%)	BT1A; BT 2, O:5; BT4, O:3	
	Colon contents: piglets		3/35 (8.6%)		
	Tonsils: finishing pigs		4/126 (3.2%)	BT2, O:5; BT4, O:3	
	Tonsils: piglets		0/35 (0%)		
	Lymph nodes: finishing pigs		3/126 (2.8%	BT1A; BT4, O:3	
	Lymph nodes: piglets		1/35 (2.8%)		
Ireland	Rectal and environmental swabs	YE	3/576 (0.52%)	BT2, O:9; BT1A	[122]
	Rectal swab at abattoir		1/20 (5%)	BT2, O:9	
	Carcass		0/20	-	
Norway	Carcass (before chilling)	YE	6/60 (10%)	BT4, O:3; BT2, O:9	[112]
	Carcass (after chilling)		5/60 (8.3%)		
South Korea	Carcass (at slaughter)	YE	0/100 (0%)	Not reported	[123]
	Pork samples	YE	0/300 (0%)	Not reported	
Sweden	Fecal (at farm)	YE	32/105 (30.5%)	BT4, O:3; BT2, O:9	[124]

**Table 4:** *Yersinia* isolated by NZ animal health laboratories, 1988–1996 (extracted from [43]).

Animal	YE Bioserotype ^a^						YP ^a^	Total
	BT1A	BT2/3 O:9	BT2/3 O:5, 27	BT2/3 O:1, 2, 3	BT4 O:3	BT5 O:2, 3		
Cattle	7	25	21			34	17	104
Sheep	4	3	1	2		77	1	88
Goats			3			55		58
Deer	12	9	9			5		35
Alpaca	3	1	4			3		11
Dogs	5	4	9		6		2	26
Cats		1	2				1	4
Pigs	3		1					4
Horses	3	1	1					5
Birds	5						7	12
Total	42	44	51	2	6	174	28	347

^a^*Yersinia enterocolitica* (YE), biotype (BT) and *Y. pseudotuberculosis* (YP).

**Table 5:** *Yersinia* typed from New Zealand animal health laboratories, January–March 2018.

Animal	YE BT2/3 O:9 ST12 ^a^	YE BT5 O:3 ^b^ ST13 ^a^	YP ST19 ^a^	YP ST43 ^a^	Total
Cattle	1	2	87		90
Unspecified			2		2
Budgerigar				1	1
**Total**	1	2	89	1	93

^a^*Yersinia enterocolitica* (YE), biotype (BT), and *Y. pseudotuberculosis* (YP). Multilocus sequence type (ST) of YE and YP was inferred using whole-genome sequencing data and using schemes of [38] and [64], respectively. ^b^ O:2 antiserum not available for this study.

## Data Availability

The data presented in this study are available on request from the corresponding author.

## References

[1] Centre for Disease Control and Prevention (CDC) *Yersinia enterocolitica* (Yersiniosis). https://www.cdc.gov/yersinia/healthcare.html.

[2] New Zealand Ministry of Health Yersiniosis. https://www.health.govt.nz/our-work/diseases-and-conditions/communicable-disease-control-manual/yersiniosis#_ftn2.

[3] Fredriksson-Ahomaa M., Korkeala H. (2003). Low occurrence of pathogenic *Yersinia enterocolitica* in clinical, food, and environmental samples: A methodological problem. Clin. Microbiol. Rev..

[4] The Institute of Environmental Science and Research Public Health Surveillance—Annual Surveillance Summary. https://surv.esr.cri.nz/surveillance/annual_surveillance.php.

[5] Williamson D.A., Baines S.L., Carter G.P., da Silva A.G., Ren X., Sherwood J., Dufour M., Schultz M.B., French N.P., Seemann T. (2016). Genomic insights into a sustained national outbreak of *Yersinia pseudotuberculosis*. Genome Biol. Evol..

[6] European Centre for Disease Prevention and Control (2019). Yersiniosis; ECDC: Stockholm. https://www.ecdc.europa.eu/sites/default/files/documents/AER_for_2018-yersiniosis-corrected.pdf.

[7] Tack D.M., Ray L., Griffin P.M., Cieslak P.R., Dunn J., Rissman T., Jervis R., Lathrop S., Muse A., Duwell M. (2020). Preliminary incidence and trends of infections with pathogens transmitted commonly through food—foodborne diseases active surveillance network, 10 U.S. Sites, 2016–2019. MMWR Morb. Mortal. Wkly. Rep..

[8] OzFoodNet Working Group (2005). Reported foodborne illness and gastroenteritis in Australia: Annual report of the OzFoodNet network, 2004. Commun. Dis. Intell..

[9] Stats NZ 2018 Census. https://www.stats.govt.nz/2018-census/.

[10] Stats NZ Ethnicity. https://www.stats.govt.nz/topics/ethnicity.

[11] The Institute of Environmental Science and Research Ltd (2020). Notifiable Diseases in New Zealand: Annual Report 2018.

[12] Chakraborty A., Komatsu K., Roberts M., Collins J., Beggs J., Turabelidze G., Safranek T., Maillard J.M., Bell L.J., Young D. (2015). The descriptive epidemiology of yersiniosis: A multistate study, 2005–2011. Public Health Rep..

[13] Long C., Jones T.F., Vugia D.J., Scheftel J., Strockbine N., Ryan P., Shiferaw B., Tauxe R.V., Gould L.H. (2010). *Yersinia pseudotuberculosis* and *Y. enterocolitica* infections, FoodNet, 1996–2007. Emerg. Infect. Dis..

[14] Lake R.J., Cressey P.J., Campbell D.M., Oakley E. (2010). Risk ranking for foodborne microbial hazards in New Zealand: Burden of disease estimates. Risk Anal..

[15] Porter C.K., Choi D., Cash B., Pimentel M., Murray J., May L., Riddle M.S. (2013). Pathogen-specific risk of chronic gastrointestinal disorders following bacterial causes of foodborne illness. BMC Gastroenterol..

[16] Highton J., Priest D. (1996). Reactive arthritis: Characteristics in southern New Zealand. N. Z. Med. J..

[17] Rosner B.M., Werber D., Höhle M., Stark K. (2013). Clinical aspects and self-reported symptoms of sequelae of *Yersinia enterocolitica* infections in a population-based study, Germany 2009–2010. BMC Infect. Dis..

[18] Hannu T., Mattila L., Nuorti J.P., Ruutu P., Mikkola J., Siitonen A., Leirisalo-Repo M. (2003). Reactive arthritis after an outbreak of *Yersinia pseudotuberculosis* serotype O:3 infection. Ann. Rheum. Dis..

[19] Stolk-Engelaar V.M., Hoogkamp-Korstanje J.A. (1996). Clinical presentation and diagnosis of gastrointestinal infections by *Yersinia enterocolitica* in 261 Dutch patients. Scand. J. Infect. Dis..

[20] Inman R.D., Chiu B., Johnston M.E., Falk J. (1986). Molecular mimicry in Reiter’s syndrome: Cytotoxicity and ELISA studies of *HLA*-microbial relationships. Immunology.

[21] Sheehan N.J. (2004). The ramifications of *HLA-B27*. J. R. Soc. Med..

[22] Roberts R.L., Wallace M.C., Jones G.T., van Rij A.M., Merriman T.R., Harrison A., White D., Stamp L.K., Ching D., Highton J. (2013). Prevalence of *HLA-B27* in the New Zealand population: Effect of age and ethnicity. Arthritis Res. Ther..

[23] Bancerz-Kisiel A., Szweda W. (2015). Yersiniosis—a zoonotic foodborne disease of relevance to public health. Ann. Agric. Environ. Med..

[24] Hugot J.P., Dumay A., Barreau F., Meinzer U. (2020). Crohn’s disease: Is the cold chain hypothesis still hot?. J. Crohns Colitis.

[25] Le Baut G., O’Brien C., Pavli P., Roy M., Seksik P., Tréton X., Nancey S., Barnich N., Bezault M., Auzolle C. (2018). Prevalence of *Yersinia* species in the ileum of Crohn’s disease patients and controls. Front. Cell Infect. MicroBiol..

[26] Effraimidis G., Tijssen J.G.P., Strieder T.G.A., Wiersinga W.M. (2011). No causal relationship between Yersinia enterocolitica infection and autoimmune thyroid disease: Evidence from a prospective study. Clin. Exp. Immunol..

[27] Bottone E.J. (1997). *Yersinia enterocolitica*: The charisma continues. Clin. MicroBiol. Rev..

[28] Horinouchi T., Nozu K., Hamahira K., Inaguma Y., Abe J., Nakajima H., Kugo M., Iijima K. (2015). *Yersinia pseudotuberculosis* infection in Kawasaki disease and its clinical characteristics. BMC Pediatr..

[29] Majowicz S.E., Panagiotoglou D., Taylor M., Gohari M.R., Kaplan G.G., Chaurasia A., Leatherdale S.T., Cook R.J., Patrick D.M., Ethelberg S. (2020). Determining the long-term health burden and risk of sequelae for 14 foodborne infections in British Columbia, Canada: Protocol for a retrospective population-based cohort study. BMJ Open.

[30] Pogreba-Brown K., Austhof E., Armstrong A., Schaefer K., Villa Zapata L., McClelland D.J., Batz M.B., Kuecken M., Riddle M., Porter C.K. (2020). Chronic gastrointestinal and joint-related sequelae associated with common foodborne illnesses: A scoping review. Foodborne Pathog. Dis..

[31] Nicol C., King N., Pirie R., Dufour M. (2010). Diagnostic and Public Health Management Practices of Foodborne Bacterial Diseases.

[B32-pathogens-10-00191] 32.Addidle, M. Clinical Microbiologist, New Zealand Microbiology Network Liaison., Email Communication with J. Wright, December 2020

[33] International Organization for Standardization (ISO) (2012). ISO15189—Medical Laboratories—Requirements for Quality and Competence. https://www.iso.org/obp/ui/#iso:std:iso:15189:ed-3:v2:en.

[34] King G. (2017). An Outbreak of Yersiniosis in Tauranga during October and November 2016.

[35] Paixão R., Moreno L.Z., Sena de Gobbi D.D., Raimundo D.C., Hofer E., Matté M.H., Ferreira T.S.P., de Moura Gomes V.T., Costa B.L.P., Moreno A.M. (2013). Characterization of *Yersinia enterocolitica* biotype 1A strains isolated from swine slaughterhouses and markets. Sci. World J..

[36] Carniel E., Guilvout I., Prentice M. (1996). Characterization of a large chromosomal “high-pathogenicity island” in biotype 1B *Yersinia enterocolitica*. J. Bacteriol..

[37] Wauters G., Kandolo K., Janssens M. (1987). Revised biogrouping scheme of *Yersinia enterocolitica*. Contrib. MicroBiol. Immunol..

[38] Hall M., Chattaway M.A., Reuter S., Savin C., Strauch E., Carniel E., Connor T., Van Damme I., Rajakaruna L., Rajendram D. (2015). Use of whole-genus genome sequence data to develop a multilocus sequence typing tool that accurately identifies *Yersinia* isolates to the species and subspecies levels. J. Clin. MicroBiol..

[39] Strydom H., Wang J., Paine S., Dyet K., Cullen K., Wright J. (2019). Evaluating sub-typing methods for pathogenic *Yersinia enterocolitica* to support outbreak investigations in New Zealand. Epidemiol. Infect..

[40] Reuter S., Connor T.R., Barquist L., Walker D., Feltwell T., Harris S.R., Fookes M., Hall M.E., Petty N.K., Fuchs T.M. (2014). Parallel independent evolution of pathogenicity within the genus *Yersinia*. Proc. Natl. Acad. Sci. USA.

[41] Eksić S., Steigerwalt A.G., Bockemühl J., Huntley-Carter G.P., Brenner D.J. (1987). *Yersinia rohdei* sp. nov. isolated from human and dog feces and surface water. Int. J. Syst. Evol..

[42] Hilbink F., Fenwick S., Thompson E.J., Penrose M., Ross G.P. (1995). Non-specific seroreactions against *Brucella abortus* in ruminants in New Zealand and the presence of *Yersinia enterocolitica* O:9. N. Z. Vet. J..

[43] Fenwick S. (1997). Domestic animals as potential sources of human *Yersinia* infection. Surveillance.

[B44-pathogens-10-00191] 44.Staples, M. Senior Scientist, Reference Laboratory, Public Health Microbiology, Forensic and Scientific Services, Queensland Health, Australia November 2020, Email Communication with J. Wright, December 2020

[45] Campioni F., Falcao J.P. (2014). Genotyping of *Yersinia enterocolitica* biotype 1A strains from clinical and nonclinical origins by pulsed-field gel electrophoresis. Can. J. MicroBiol..

[46] Sihvonen L.M., Jalkanen K., Huovinen E., Toivonen S., Corander J., Kuusi M., Skurnik M., Siitonen A., Haukka K. (2012). Clinical isolates of *Yersinia enterocolitica* biotype 1A represent two phylogenetic lineages with differing pathogenicity-related properties. BMC MicroBiol..

[47] Tennant S.M., Grant T.H., Robins-Browne R.M. (2003). Pathogenicity of *Yersinia enterocolitica* biotype 1A. FEMS Immunol. Med. MicroBiol..

[48] Tuompo R., Hannu T., Huovinen E., Sihvonen L., Siitonen A., Leirisalo-Repo M. (2017). *Yersinia enterocolitica* biotype 1A: A possible new trigger of reactive arthritis. Rheumatol.Int..

[49] Grant T., Bennett-Wood V., Robins-Browne R.M. (1998). Identification of virulence-associated characteristics in clinical isolates of *Yersinia enterocolitica* lacking classical virulence markers. Infect. Immun..

[50] Ramamurthy T., Yoshino K., Huang X., Balakrish Nair G., Carniel E., Maruyama T., Fukushima H., Takeda T. (1997). The novel heat-stable enterotoxin subtype gene (*ystB*) of *Yersinia enterocolitica*: Nucleotide sequence and distribution of the *yst* genes. Microb. Pathog..

[51] Stephan R., Joutsen S., Hofer E., Säde E., Björkroth J., Ziegler D., Fredriksson-Ahomaa M. (2013). Characteristics of *Yersinia enterocolitica* biotype 1A strains isolated from patients and asymptomatic carriers. Eur. J. Clin. MicroBiol. Infect. Dis..

[52] Wojciech Ł., Staroniewicz Z., Jakubczak A., Ugorski M. (2004). Typing of *Yersinia enterocolitica* isolates by ITS profiling, REP- and ERIC-PCR. J. Vet. Med. B.

[53] Gilpin B.J., Robson B., Lin S., Hudson J.A., Weaver L., Dufour M., Strydom H. (2014). The limitations of pulsed-field gel electrophoresis for analysis of *Yersinia enterocolitica* isolates. Zoonoses Public Health.

[54] Gierczyński R., Golubov A., Neubauer H., Pham J.N., Rakin A. (2007). Development of multiple-locus variable-number tandem-repeat analysis for *Yersinia enterocolitica* subsp. *palearctica* and its application to bioserogroup 4/O3 subtyping. J. Clin. MicroBiol..

[55] Gulati P., Varshney R.K., Virdi J.S. (2009). Multilocus variable number tandem repeat analysis as a tool to discern genetic relationships among strains of *Yersinia enterocolitica* biovar 1A. J. Appl. MicroBiol..

[56] Besser J.M., Carleton H.A., Trees E., Stroika S.G., Hise K., Wise M., Gerner-Smidt P. (2019). Interpretation of whole-genome sequencing for enteric disease surveillance and outbreak investigation. Foodborne Pathog. Dis..

[57] Inns T., Flanagan S., Greig D.R., Jenkins C., Seddon K., Chin T., Cartwright J. (2018). First use of whole-genome sequencing to investigate a cluster of *Yersinia enterocolitica*, Liverpool, United Kingdom, 2017. J. Med. MicroBiol..

[58] Food and Agriculture Organization of the United Nations (2016). Technical Background Paper: Applications of Whole Genome Sequencing in Food Safety Management.

[59] Seecharran T., Kalin-Mänttäri L., Koskela K.A., Nikkari S., Dickins B., Corander J., Skurnik M., McNally A. (2017). Phylogeographic separation and formation of sexually discrete lineages in a global population of *Yersinia pseudotuberculosis*. bioRxiv.

[60] Savin C., Criscuolo A., Guglielmini J., Le Guern A.S., Carniel E., Pizarro-Cerdá J., Brisse S. (2019). Genus-wide *Yersinia* core-genome multilocus sequence typing for species identification and strain characterization. Microb. Genom..

[61] Zhou Z., Charlesworth J., Achtman M. (2020). HierCC: A multi-level clustering scheme for population assignments based on core genome MLST. bioRxiv.

[62] Zhou Z., Alikhan N.-F., Mohamed K., Fan Y., Agama Study G., Achtman M. (2020). The EnteroBase user’s guide, with case studies on *Salmonella* transmissions, *Yersinia pestis* phylogeny, and *Escherichia* core genomic diversity. Genome Res..

[63] Seemann T., Goncalves da Silva A., Bulach D.M., Schultz M.B., Kwong J.C., Howden B.P. Nullarbor GitHub. https://github.com/tseemann/nullarbor.

[64] Laukkanen-Ninios R., Didelot X., Jolley K.A., Morelli G., Sangal V., Kristo P., Brehony C., Imori P.F.M., Fukushima H., Siitonen A. (2011). Population structure of the *Yersinia pseudotuberculosis* complex according to multilocus sequence typing. Environ. MicroBiol..

[65] Seemann T., Goncalves da Silva A., Bulach D.M., Schultz M.B., Kwong J.C., Howden B.P. Snippy, Rapid Bacterial SNP Calling and Core Genome Alignments. https://github.com/tseemann/snippy.

[66] Dallman T., Ashton P., Schafer U., Jironkin A., Painset A., Shaaban S., Hartman H., Myers R., Underwood A., Jenkins C. (2018). SnapperDB: A database solution for routine sequencing analysis of bacterial isolates. Bioinformatics.

[67] Argimón S., Abudahab K., Goater R.J.E., Fedosejev A., Bhai J., Glasner C., Feil E.J., Holden M.T.G., Yeats C.A., Grundmann H. (2016). Microreact: Visualizing and sharing data for genomic epidemiology and phylogeography. Microb. Genom..

[68] Nguyen L.-T., Schmidt H.A., von Haeseler A., Minh B.Q. (2014). IQ-TREE: A fast and effective stochastic algorithm for estimating maximum-likelihood phylogenies. Mol. Biol. Evol..

[69] Hoang D.T., Chernomor O., von Haeseler A., Minh B.Q., Vinh L.S. (2017). UFBoot2: Improving the ultrafast bootstrap approximation. Mol. Biol. Evol..

[70] Lake R., Hudson A., Cressey P. (2004). Risk Profile: Yersinia enterocolitica in Pork.

[71] Satterthwaite P., Pritchard K., Floyd D., Law B. (1999). A case control study of *Yersinia enterocolitica* infections in Auckland. Aust. N. Z. J. Publ. Health.

[72] Wright J. (1996). Gastrointestinal Infection in a New Zealand Community: A One Year Study.

[73] New Zealand Ministry of Health Appendix 2: Enteric Disease. https://www.health.govt.nz/our-work/diseases-and-conditions/communicable-disease-control-manual/appendix-2-enteric-disease.

[74] Cressey P.J., Lake R.J., Thornley C., Campbell D. (2019). Expert elicitation for estimation of the proportion foodborne for selected microbial pathogens in New Zealand. Foodborne Pathog. Dis..

[75] Zanabria R., Racicot M., Leroux A., Xucen L., Cormier M., Ferrouillet C., Arsenault J., Mackay A., Griffiths M., Holley R. (2019). Source attribution at the food sub-product level for the development of the Canadian Food Inspection Agency risk assessment model. Int. J. Food MicroBiol..

[76] Scallan E., Hoekstra R.M., Angulo F.J., Tauxe R.V., Widdowson M.-A., Roy S.L., Jones J.L., Griffin P.M. (2011). Foodborne illness acquired in the United States--major pathogens. Emerg. Infect. Dis..

[77] Butler A.J., Thomas M.K., Pintar K.D. (2015). Expert elicitation as a means to attribute 28 enteric pathogens to foodborne, waterborne, animal contact, and person-to-person transmission routes in Canada. Foodborne Pathog. Dis..

[78] Adak G.K., Long S.M., O’Brien S.J. (2002). Trends in indigenous foodborne disease and deaths, England and Wales: 1992 to 2000. Gut.

[79] New Zealand Ministry for Primary Industries (2014). Outbreak Source Investigation: Yersinia pseudotuberculosis 2014.

[80] Espenhain L., Riess M., Muller L., Colombe S., Ethelberg S., Litrup E., Jernberg C., Kuhlmann-Berenzon S., Lindblad M., Hove N.K. (2019). Cross-border outbreak of *Yersinia enterocolitica* O:3 associated with imported fresh spinach, Sweden and Denmark, March 2019. Euro Surveill.

[81] Minami K., Yasuda R., Terakawa R., Koike Y., Takeuchi K., Higuchi T., Horiuchi A., Kubota N., Hidaka E., Kawakami Y. (2017). Four sporadic pediatric cases of *Yersinia enterocolitica* O:8 infection in a rural area of Japan. Jpn. J. Infect. Dis..

[82] MacDonald E., Einoder-Moreno M., Borgen K., Thorstensen Brandal L., Diab L., Fossli O., Guzman Herrador B., Hassan A.A., Johannessen G.S., Johansen E.J. (2016). National outbreak of *Yersinia enterocolitica* infections in military and civilian populations associated with consumption of mixed salad, Norway, 2014. Euro Surveill..

[83] MacDonald E., Heier B.T., Stalheim T., Cudjoe K.S., Skjerdal T., Wester A., Lindstedt B.A., Vold L. (2011). *Yersinia enterocolitica* O:9 infections associated with bagged salad mix in Norway, February to April 2011. Euro Surveill..

[84] Center for Disease Control and Prevention (CDC) (2011). Notes from the field: *Yersinia enterocolitica* infections associated with pasteurized milk—southwestern Pennsylvania, March-August, 2011. MMWR Morb. Mortal. Mortal. Wkly. Rep..

[85] OzFoodNet (2009). Communicable Disease Intelligence-Quarterly Report.

[86] Grahek-Ogden D., Schimmer B., Cudjoe K.S., Nygard K., Kapperud G. (2007). Outbreak of *Yersinia enterocolitica* serogroup O:9 infection and processed pork, Norway. Emerg. Infect. Dis..

[87] Sakai T., Nakayama A., Hashida M., Yamamoto Y., Takebe H., Imai S. (2005). Outbreak of food poisoning by *Yersinia enterocolitica* serotype O8 in Nara prefecture: The first case report in Japan. Jpn. J. Infect. Dis..

[88] Centre for Disease Control and Prevention (CDC) (2003). *Yersinia enterocolitica* gastroenteritis among infants exposed to chitterlings—Chicago, Illinois, 2002. MMWR Morb. Mortal. Mortal. Wkly. Rep..

[89] Pärn T., Hallanvuo S., Salmenlinna S., Pihlajasaari A., Heikkinen S., Telkki-Nykänen H., Hakkinen M., Ollgren J., Huusko S., Rimhanen-Finne R. (2015). Outbreak of *Yersinia pseudotuberculosis* O:1 infection associated with raw milk consumption, Finland, spring 2014. Euro Surveill..

[90] Rimhanen-Finne R., Niskanen T., Hallanvuo S., Makary P., Haukka K., Pajunen S., Siitonen A., Ristolainen R., Poyry H., Ollgren J. (2008). *Yersinia pseudotuberculosis* causing a large outbreak associated with carrots in Finland, 2006. Epidemiol. Infect..

[91] Kangas S., Takkinen J., Hakkinen M., Nakari U.M., Johansson T., Henttonen H., Virtaluoto L., Siitonen A., Ollgren J., Kuusi M. (2008). *Yersinia pseudotuberculosis* O:1 traced to raw carrots, Finland. Emerg. Infect. Dis..

[92] Jalava K., Hakkinen M., Valkonen M., Nakari U.M., Palo T., Hallanvuo S., Ollgren J., Siitonen A., Nuorti J.P. (2006). An outbreak of gastrointestinal illness and erythema nodosum from grated carrots contaminated with *Yersinia pseudotuberculosis*. J. Infect. Dis..

[93] Boqvist S., Pettersson H., Svensson A., Andersson Y. (2009). Sources of sporadic *Yersinia enterocolitica* infection in children in Sweden, 2004: A case-control study. Epidemiol. Infect..

[94] Ostroff S.M., Kapperud G., Hutwagner L.C., Nesbakken T., Bean N.H., Lassen J., Tauxe R.V. (1994). Sources of sporadic *Yersinia enterocolitica* infections in Norway: A prospective case-control study. Epidemiol. Infect..

[95] Jones T.F., Buckingham S.C., Bopp C.A., Ribot E., Schaffner W. (2003). From pig to pacifier: Chitterling-associated yersiniosis outbreak among black infants. Emerg. Infect. Dis..

[96] Guillier L., Fravalo P., Leclercq A., Thébault A., Kooh P., Cadavez V., Gonzales-Barron U. (2020). Risk factors for sporadic *Yersinia enterocolitica* infections: A systematic review and meta-analysis. Microbial. Risk Anal..

[97] MacDonald E., Heier B.T., Nygard K., Stalheim T., Cudjoe K.S., Skjerdal T., Wester A.L., Lindstedt B.A., Stavnes T.L., Vold L. (2012). *Yersinia enterocolitica* outbreak associated with ready-to-eat salad mix, Norway, 2011. Emerg. Infect. Dis..

[98] Tauxe R.V., Wauters G., Goossens V., Noyen R.V., Vandepitte J., Martin S.M., Mol P.D., Thiers G. (1987). *Yersinia enterocolitica* infections and pork: The missing link. Lancet.

[99] Huovinen E., Sihvonen L.M., Virtanen M.J., Haukka K., Siitonen A., Kuusi M. (2010). Symptoms and sources of *Yersinia enterocolitica*-infection: A case-control study. BMC Infect. Dis..

[100] Rosner B.M., Stark K., Höhle M., Werber D. (2012). Risk factors for sporadic *Yersinia enterocolitica* infections, Germany 2009–2010. Epidemiol. Infect..

[101] Nuorti J.P., Niskanen T., Hallanvuo S., Mikkola J., Kela E., Hatakka M., Fredriksson-Ahomaa M., Lyytikainen O., Siitonen A., Korkeala H. (2004). A widespread outbreak of *Yersinia pseudotuberculosis* O:3 infection from iceberg lettuce. J. Infect. Dis..

[102] Jalava K., Hallanvuo S., Nakari U.M., Ruutu P., Kela E., Heinasmaki T., Siitonen A., Nuorti J.P. (2004). Multiple outbreaks of *Yersinia pseudotuberculosis* infections in Finland. J. Clin. MicroBiol..

[103] Fenwick S. (1997). Yersinia enterocolitica Infections in People and Other Animals—A New Zealand Study.

[104] Hudson J.A., Mott S.J., Delacy K.M., Edridge A.L. (1992). Incidence and coincidence of *Listeria* spp., motile aeromonads and *Yersinia enterocolitica* on ready-to-eat fleshfoods. Int. J. Food Microbiol..

[105] Laukkanen-Ninios R., Fredriksson-Ahomaa M., Korkeala H. (2014). Enteropathogenic *Yersinia* in the pork production chain: Challenges for control. Compr. Rev. Food Sci. Food Saf..

[106] Drummond N., Murphy B.P., Ringwood T., Prentice M.B., Buckley J.F., Fanning S. (2012). *Yersinia enterocolitica*: A brief review of the issues relating to the zoonotic pathogen, public health challenges, and the pork production chain. Foodborne Pathog. Dis..

[107] Messelhausser U., Kampf P., Colditz J., Bauer H., Schreiner H., Holler C., Busch U. (2011). Qualitative and quantitative detection of human pathogenic *Yersinia enterocolitica* in different food matrices at retail level in Bavaria. Foodborne Pathog. Dis..

[108] Fredriksson-Ahomaa M., Hielm S., Korkeala H. (1999). High prevalence of *yadA*-positive *Yersinia enterocolitica* in pig tongues and minced meat at the retail level in Finland. J. Food Prot..

[109] Fredriksson-Ahomaa M., Lyhs U., Korte T., Korkeala H. (2001). Prevalence of pathogenic *Yersinia enterocolitica* in food samples at retail level in Finland. Archiv. Lebensm..

[110] Arrausi-Subiza M., Ibabe J.C., Atxaerandio R., Juste R.A., Barral M. (2014). Evaluation of different enrichment methods for pathogenic *Yersinia* species detection by real time PCR. BMC Vet. Res..

[111] Van Damme I., Berkvens D., Vanantwerpen G., Bare J., Houf K., Wauters G., De Zutter L. (2015). Contamination of freshly slaughtered pig carcasses with enteropathogenic *Yersinia* spp.: Distribution, quantification and identification of risk factors. Int. J. Food MicroBiol..

[112] Nesbakken T., Eckner K., Røtterud O.-J. (2008). The effect of blast chilling on occurrence of human pathogenic *Yersinia enterocolitica* compared to *Campylobacter* spp. and numbers of hygienic indicators on pig carcasses. Int. J. Food MicroBiol..

[113] Van Damme I., De Zutter L., Jacxsens L., Nauta M.J. (2017). Control of human pathogenic *Yersinia enterocolitica* in minced meat: Comparative analysis of different interventions using a risk assessment approach. Food MicroBiol..

[114] Martins B.T.F., Botelho C.V., Silva D.A.L., Lanna F., Grossi J.L., Campos-Galvão M.E.M., Yamatogi R.S., Falcão J.P., Bersot L.D.S., Nero L.A. (2018). *Yersinia enterocolitica* in a Brazilian pork production chain: Tracking of contamination routes, virulence and antimicrobial resistance. Int. J. Food MicroBiol..

[115] Liang J., Wang X., Xiao Y., Cui Z., Xia S., Hao Q., Yang J., Luo L., Wang S., Li K. (2012). Prevalence of *Yersinia enterocolitica* in pigs slaughtered in Chinese abattoirs. Appl. Environ. MicroBiol..

[116] Rahikainen Ibañez T., Laukkanen-Ninios R., Hakkinen M., Johansson T., Vilar M., Korkeala H. (2016). Prevalence of pathogenic *Yersinia enterocolitica* in Finnish slaughter pigs. J. Food Prot..

[117] Fondrevez M., Minvielle B., Labbé A., Houdayer C., Rose N., Esnault E., Denis M. (2014). Prevalence of pathogenic *Yersinia enterocolitica* in slaughter-aged pigs during a one-year survey, 2010-2011, France. Int. J. Food MicroBiol..

[118] Gürtler M., Alter K., Kasimir S., Linnebur M., Fahlhaber K. (2005). Prevalence of *Yersinia enterocolitica* in fattening pigs. J. Food Prot..

[119] Bonardi S., Bassi L., Brindani F., D’Incau M., Barco L., Carra E., Pongolini S. (2013). Prevalence, characterization and antimicrobial susceptibility of *Salmonella enterica* and *Yersinia enterocolitica* in pigs at slaughter in Italy. Int. J. Food MicroBiol..

[120] Bonardi S., Bruini I., D’Incau M., Van Damme I., Carniel E., Brémont S., Cavallini P., Tagliabue S., Brindani F. (2016). Detection, seroprevalence and antimicrobial resistance of *Yersinia enterocolitica* and *Yersinia pseudotuberculosis* in pig tonsils in Northern Italy. Int. J. Food MicroBiol..

[121] Fois F., Piras F., Torpdahl M., Mazza R., Ladu D., Consolati S.G., Spanu C., Scarano C., De Santis E.P.L. (2018). Prevalence, bioserotyping and antibiotic resistance of pathogenic *Yersinia enterocolitica* detected in pigs at slaughter in Sardinia. Int. J. Food MicroBiol..

[122] Bolton D.J., Ivory C., McDowell D. (2013). A small study of *Yersinia enterocolitica* in pigs from birth to carcass and characterisation of porcine and human strains. Food Control..

[123] Choi Y.M., Park H.J., Jang H.I., Kim S.A., Imm J.Y., Hwang I.G., Rhee M.S. (2013). Changes in microbial contamination levels of porcine carcasses and fresh pork in slaughterhouses, processing lines, retail outlets, and local markets by commercial distribution. Res. Vet. Sci..

[124] Råsbäck T., Rosendal T., Stampe M., Sannö A., Aspán A., Järnevi K., Lahti E.T. (2018). Prevalence of human pathogenic *Yersinia enterocolitica* in Swedish pig farms. Acta Vet. Scand..

[125] Syczyło K., Platt-Samoraj A., Bancerz-Kisiel A., Szczerba-Turek A., Pajdak-Czaus J., Łabuć S., Procajło Z., Socha P., Chuzhebayeva G., Szweda W. (2018). The prevalence of *Yersinia enterocolitica* in game animals in Poland. PLoS ONE.

[126] Sannö A., Aspán A., Hestvik G., Jacobson M. (2014). Presence of *Salmonella* spp., *Yersinia enterocolitica*, *Yersinia pseudotuberculosis* and *Escherichia coli* O157:H7 in wild boars. Epidemiol. Infect..

[127] Backhans A., Fellström C., Lambertz S.T. (2011). Occurrence of pathogenic *Yersinia enterocolitica* and *Yersinia pseudotuberculosis* in small wild rodents. Epidemiol. Infect..

[128] Reinhardt M., Hammerl J.A., Kunz K., Barac A., Nöckler K., Hertwig S. (2018). *Yersinia pseudotuberculosis* prevalence and diversity in wild boars in Northeast Germany. Appl. Environ. MicroBiol..

[129] Niskanen T., Waldenström J., Fredriksson-Ahomaa M., Olsen B., Korkeala H. (2003). *virF*-positive *Yersinia pseudotuberculosis* and *Yersinia enterocolitica* found in migratory birds in Sweden. Appl. Environ. MicroBiol..

[130] Arrausi-Subiza M., Gerrikagoitia X., Alvarez V., Ibabe J.C., Barral M. (2016). Prevalence of *Yersinia enterocolitica* and *Yersinia pseudotuberculosis* in wild boars in the Basque Country, northern Spain. Acta Vet. Scand..

[131] Fredriksson-Ahomaa M., Wacheck S., Bonke R., Stephan R. (2011). Different enteropathogenic *Yersinia* strains found in wild boars and domestic pigs. Foodborne Pathog. Dis..

[132] Niskanen T., Fredriksson-Ahomaa M., Korkeala H., Skurnik M., Bengoechea J.A., Granfors K. (2003). Occurence of *Yersinia pseudotuberculosis* in iceberg lettuce and environment. The Genus Yersinia: Entering the Functional Genomic Era.

[133] Pattis I., Moriarty E., Billington C., Gilpin B., Hodson R., Ward N. (2017). Concentrations of *Campylobacter* spp., *Escherichia coli, Enterococci*, and *Yersinia* spp. in the feces of farmed red deer in New Zealand. J. Environ. Qual..

[134] Wang X., Cui Z., Wang H., Tang L., Yang J., Gu L., Jin D., Luo L., Qiu H., Xiao Y. (2010). Pathogenic strains of *Yersinia enterocolitica* isolated from domestic dogs (*Canis familiaris*) belonging to farmers are of the same subtype as pathogenic *Y. enterocolitica* strains isolated from humans and may be a source of human infection in Jiangsu Province, China. J. Clin. MicroBiol..

[135] Fredriksson-Ahomaa M., Korte T., Korkeala H. (2001). Transmission of *Yersinia enterocolitica* 4/O:3 to pets via contaminated pork. Lett. Appl. MicroBiol..

[136] Le Guern A.-S., Martin L., Savin C., Carniel E. (2016). Yersiniosis in France: Overview and potential sources of infection. Int. J. Infect. Dis..

[137] Isobe J., Kimata K., Shimizu M., Kanatani J., Sata T., Watahiki M. (2014). Water-borne outbreak of *Yersinia enterocolitica* O8 due to a small scale water system. Kansenshogaku Zasshi.

[138] Eden K.V., Rosenberg M.L., Stoopler M., Wood B.T., Highsmith A.K., Skaliy P., Wells J.G., Feeley J.C. (1977). Waterborne gastrointestinal illness at a ski resort. —Isolation of *Yersinia enterocolitica* from drinking water. Public Health Rep..

[139] New Zealand Ministry of Health (2018). Drinking-Water Standards for New Zealand 2005 (Revised 2018).

[140] Sandery M., Stinear T., Kaucner C. (1996). Detection of pathogenic *Yersinia enterocolitica* in environmental waters by PCR. J. Appl. MicroBiol..

[141] Falcão J.P., Brocchi M., Proença-Módena J.L., Acrani G.O., Corrêa E.F., Falcão D.P. (2004). Virulence characteristics and epidemiology of *Yersinia enterocolitica* and Yersiniae other than *Y. pseudotuberculosis* and *Y. pestis* isolated from water and sewage. J. Appl. MicroBiol..

[142] Moriki S., Nobata A., Shibata H., Nagai A., Minami N., Taketani T., Fukushima H. (2010). Familial outbreak of *Yersinia enterocolitica* serotype O9 biotype 2. J. Infect. Chemther..

[143] Morse D.L., Shayegani M., Gallo R.J. (1984). Epidemiologic investigation of a *Yersinia* camp outbreak linked to a food handler. Am. J. Public Health.

[144] Ratnam S., Mercer E., Picco B., Parsons S., Butler R. (1982). A nosocomial outbreak of diarrheal disease due to *Yersinia enterocolitica* serotype O:5, biotype 1. J. Infect. Dis..

[145] Theakston E.P., Baker B.W., Morris A.J., Woodfield D.G., Streat S.J. (1997). Transfusion transmitted *Yersinia enterocolitica* infections in New Zealand. Aust. N. Z. Med. J..

[B146-pathogens-10-00191] 146.Morley, S. Chief Medical Officer, NZBlood, Email Communication with J. Wright, December 2020

[147] New Zealand Blood (2010). Annual Report 2010.

[B148-pathogens-10-00191] 148.Watts, J. Senior Adviser Animal Health Surveillance and Incursion Investigation Biosecurity New Zealand Ministry for Primary Industries, Email Communication with J. Wright, December 2020

[149] Mair N.S., Fox E., Thal E. (1979). Biochemical, pathogenicity and toxicity studies of type III strains of *Yersinia pseudotuberculosis* isolated from the cecal contents of pigs. Contrib. MicroBiol. Immunol..

[150] Tsubokura M., Otsuki K., Kawaoka Y., Maruyama T. (1984). Characterization and pathogenicity of *Yersinia pseudotuberculosis* isolated from swine and other animals. J. Clin. MicroBiol..

[151] Martins C.H., Bauab T.M., Falcão D.P. (1998). Characteristics of *Yersinia pseudotuberculosis* isolated from animals in Brazil. J. Appl. MicroBiol..

[152] Buhles W.C., Vanderlip J.E., Russell S.W., Alexander N.L. (1981). *Yersinia pseudotuberculosis* infection: Study of an epizootic in squirrel monkeys. J. Clin. MicroBiol..

[153] Warth J.F., Biesdorf S.M., de Souza C. (2012). *Yersinia pseudotuberculosis* O III causes diarrhea in Brazilian cattle. Adv. Exp. Med. Biol..

[154] Aleksić S., Bockemühl J., Wuthe H.H. (1995). Epidemiology of *Y. pseudotuberculosis* in Germany, 1983–1993. Contrib. MicroBiol. Immunol..

[155] EnteroBase Yersinia.

[156] Gupta V., Gulati P., Bhagat N., Dhar M.S., Virdi J.S. (2015). Detection of *Yersinia enterocolitica* in food: An overview. Eur. J. Clin. MicroBiol. Infect. Dis..

[157] Fukushima H., Shimizu S., Inatsu Y. (2011). *Yersinia enterocolitica* and *Yersinia pseudotuberculosis* detection in foods. J. Pathog..

[158] International Organization for Standardization (ISO) (2017). ISO/FDIS. 10273—Microbiology of the Food Chain—Horizontal Method for the Detection of Pathogenic Yersinia enterocolitica. https://infostore.saiglobal.com/en-us/Standards/ISO-10273-2017-588078_SAIG_ISO_ISO_1347019/.

[159] European Centre for Disease Prevention and Control (EFSA) (2009). Technical specifications for harmonised national surveys of *Yersinia enterocolitica* in slaughtered pigs. EFSA J..

[160] Van Damme I., Habib I., De Zutter L. (2010). *Yersinia enterocolitica* in slaughter pig tonsils: Enumeration and detection by enrichment versus direct plating culture. Food MicroBiol..

[161] Hallanvuo S., Herranen M., Jaakkonen A., Nummela M., Ranta J., Botteldoorn N., De Zutter L., Fredriksson-Ahomaa M., Hertwig S., Johannessen G.S. (2018). Validation of ISO method 10273—Detection of pathogenic *Yersinia enterocolitica* in foods. Int. J. Food MicroBiol..

[162] Wauters G., Goossens V., Janssens M., Vandepitte J. (1988). New enrichment medium for isolation of pathogenic *Yersinia enterocolitica* serogroup O:3 in pork. Appl. Environ. MicroBiol..

[163] Weagant S.D., Feng P. Yersinia enterocolitica.

[164] Fukushima H., Gomyoda M. (1986). Growth of *Yersinia pseudotuberculosis* and *Yersinia enterocolitica* biotype 3B serotype O3 inhibited on cefsulodin-Irgasan-novobiocin agar. J. Clin. MicroBiol..

[165] Jourdan A.D., Johnson S.C., Wesley I.V. (2000). Development of a fluorogenic 5′ nuclease PCR assay for detection of the *ail* gene of pathogenic *Yersinia enterocolitica*. Appl. Environ. MicroBiol..

[166] Boyapalle S., Wesley I.V., Hurd H.S., Reddy P.G. (2001). Comparison of culture, multiplex, and 5′ nuclease polymerase chain reaction assays for the rapid detection of *Yersinia enterocolitica* in swine and pork products. J. Food Prot..

[167] Wu V.C.H., Fung D.Y.C., Oberst R.B. (2004). Evaluation of a 5′-nuclease (TaqMan) assay with the thin agar layer oxyrase method for detection of *Yersinia enterocolitica* in ground pork samples. J. Food Prot..

[168] Mäde D., Reiting R., Strauch E., Ketteritzsch K., Wicke A. (2008). A real-time PCR for detection of pathogenic *Yersinia enterocolitica* in food combined with an universal internal amplification control system. J. Verbr. Lebensm..

[169] Kapperud G., Vardund T., Skjerve E., Hornes E., Michaelsen T.E. (1993). Detection of pathogenic *Yersinia enterocolitica* in foods and water by immunomagnetic separation, nested polymerase chain reactions, and colorimetric detection of amplified DNA. Appl. Environ. MicroBiol..

[170] Lambertz S.T., Nilsson C., Hallanvuo S., Lindblad M. (2008). Real-time PCR method for detection of *Yersinia enterocolitica* in food. Appl. Environ. MicroBiol..

[171] Nakajima H., Inoue M., Mori T., Itoh K.-I., Arakawa E., Watanabe H. (1992). Detection and identification of *Yersinia pseudotuberculosis* and pathogenic *Yersinia enterocolitica* by an imporved polymerase chain reaction method. J. Clin. MicroBiol..

[172] Kaneko S., Ishizaki N., Kokubo Y. (1995). Detection of pathogenic *Yersinia enterocolitica* and *Yersinia pseudotuberculosis* from pork using the polymerase chain reaction. Contrib. MicroBiol. Immunol..

[173] Petsios S., Fredriksson-Ahomaa M., Sakkas H., Papadopoulou C. (2016). Conventional and molecular methods used in the detection and subtyping of *Yersinia enterocolitica* in food. Int. J. Food MicroBiol..

[174] International Organization for Standardization (ISO) (2015). ISO/TS 18867—Microbiology of the Food Chain-Polymerase Chain Reaction (PCR) for the Detection of Food-Borne Pathogens- Detection of Pathogenic Yersinia enterocolitica and Yersinia pseudotubculosi.

[175] Bonardi S., Paris A., Bassi L., Salmi F., Bacci C., Riboldi E., Boni E., D’Incau M., Tagliabue S., Brindani F. (2010). Detection, semiquantitative enumeration, and antimicrobial susceptibility of *Yersinia enterocolitica* in pork and chicken meats in Italy. J. Food Prot..

[176] Wang J.-z., Duan R., Liang J.-r., Huang Y., Xiao Y.-c., Qiu H.-y., Wang X., Jing H.-q. (2014). Real-time TaqMan PCR for *Yersinia enterocolitica* detection based on the *ail* and *foxA* genes. J. Clin. MicroBiol..

[177] Seoane A., García Lobo J.M. (1991). Cloning of chromosomal beta-lactamase genes from *Yersinia enterocolitica*. J. Gen. MicroBiol..

[178] Bonke R., Wacheck S., Stüber E., Meyer C., Märtlbauer E., Fredriksson-Ahomaa M. (2011). Antimicrobial susceptibility and distribution of β-lactamase A (*blaA*) and β-lactamase B (*blaB*) genes in enteropathogenic *Yersinia* species. Microb. Drug Resist..

[179] Saraka D., Savin C., Kouassi S., Cisse B., Koffi E., Cabanel N., Bremont S., Faye-Kette H., Dosso M., Carniel E. (2017). *Yersinia enterocolitica*, a neglected cause of human enteric infections in Cote d’Ivoire. PLoS Negl. Trop. Dis..

[180] Bhaduri S., Wesley I., Richards H., Draughon A., Wallace M. (2009). Clonality and antibiotic susceptibility of *Yersinia enterocolitica* isolated from U.S. market weight hogs. Foodborne Pathog. Dis..

[181] Lai C.-H., Lin J.-N., Chen Y.-H., Chang L.-L., Huang W.-Y., Ku H.-P., Lin H.-H. (2014). The first imported human case of *Yersinia pseudotuberculosis* serotype O1 septicemia presents with acute appendicitis-like syndrome in Taiwan. J. Formos. Med. Assoc..

[182] Magistrali C.F., Cucco L., Pezzotti G., Farneti S., Cambiotti V., Catania S., Prati P., Fabbi M., Lollai S., Mangili P. (2015). Characterisation of *Yersinia pseudotuberculosis* isolated from animals with yersiniosis during 1996–2013 indicates the presence of pathogenic and Far Eastern strains in Italy. Vet. MicroBiol..

[183] Frazão M.R., Andrade L.N., Darini A.L.C., Falcão J.P. (2017). Antimicrobial resistance and plasmid replicons in *Yersinia enterocolitica* strains isolated in Brazil in 30 years. Braz. J. Infect. Dis..

[184] Lucero-Estrada C.S., Soria J.M., Favier G.I., Escudero M.E. (2015). Evaluation of the pathogenic potential, antimicrobial susceptibility, and genomic relations of *Yersinia enterocolitica* strains from food and human origin. Can. J. MicroBiol..

[185] Stock I., Wiedemann B. (1999). An *in-vitro* study of the antimicrobial susceptibilities of *Yersinia enterocolitica* and the definition of a database. J. Antimicrob. Chemother..

[186] Health and Research Council New Zealand Unravelling the Mysteries of Yersinosis. https://hrc.govt.nz/resources/research-repository/unravelling-mysteries-yersiniosis.

